# Mitochondria-endoplasmic reticulum-lipid droplet crosstalk in lipid metabolic reprogramming in type 2 diabetes mellitus

**DOI:** 10.1016/j.jlr.2026.101146

**Published:** 2026-09-15

**Authors:** Xinyi Fang, Jiaqi Gao, Yan Wang, Danping Zhu, Jiaxing Tian, Xiaolin Tong

**Affiliations:** 1 Department of Endocrinology, Chongqing Traditional Chinese Medicine Hospital, Chongqing, China; 2 Clinical Research Center for Metabolic Diseases (Integrated Traditional Chinese and Western Medicine) of Chongqing, Chongqing, China; 3 Institute of Metabolic Diseases, Guang’anmen Hospital, China Academy of Chinese Medical Sciences, Beijing, China; 4 Graduate College, Beijing University of Chinese Medicine, Beijing, China

**Keywords:** Type 2 diabetes mellitus, lipid metabolic reprogramming, mitochondria-endoplasmic reticulum-lipid droplet interactions, membrane contact sites, mitochondria-associated ER membranes, organelle crosstalk

## Abstract

Type 2 diabetes mellitus (T2DM) is characterized by progressive metabolic reprogramming in which lipid metabolism imbalance links nutrient excess to insulin resistance, organelle stress, and tissue dysfunction. Although mitochondria, the endoplasmic reticulum (ER), and lipid droplets (LDs) each have established roles in metabolic homeostasis, how their coordinated interactions govern lipid remodeling in T2DM remains incompletely defined, owing in part to the technical difficulty of resolving these highly dynamic contact sites in space and time. In this review, we integrate current evidence on mito-ER-LD interactions in T2DM, spanning advances in omics, imaging, and biochemical approaches, and summarize their structural organization, regulatory machinery, tissue-specific remodeling, and therapeutic modulation. Emerging data identify this tri-organelle network as a central metabolic interface that couples phospholipid synthesis and trafficking, calcium flux, mitochondrial dynamics, LD biogenesis and turnover, fatty-acid oxidation, autophagy, and stress signaling. Disruption of this network, including mitochondria-associated ER membrane (MAM) instability, altered balance between cytoplasmic and peridroplet mitochondria, and pathological LD expansion, reprograms lipid handling across liver, adipose tissue, skeletal muscle, and pancreatic β cells, thereby promoting lipotoxicity, oxidative and ER stress, mitochondrial dysfunction, β-cell failure, and systemic insulin resistance. Notably, glucose-lowering drugs and lifestyle interventions may exert part of their metabolic benefit by restoring organelle crosstalk and reestablishing lipid homeostasis. Together, this review positions mito-ER-LD contact sites not simply as structural interfaces but as core nodes that regulate lipid metabolic reprogramming in T2DM, thereby providing a mechanistic framework for targeting organelle crosstalk in metabolic disease.

In recent years, research on type 2 diabetes mellitus (T2DM) has progressively moved beyond conventional concerns such as islet dysfunction, glucose homeostatic imbalance, and ectopic fat accumulation toward the elucidation of the dynamic pathological process of metabolic reprogramming (1). Among metabolically important tissues such as the liver, skeletal muscle, pancreas, and adipose tissue, a systemic disruption of lipid uptake, synthesis, storage, catabolism, transport, and oxidation is considered a central pathological link connecting nutrient excess, insulin resistance, organelle stress, and β-cell dysfunction, and lipid metabolic reprogramming has emerged as a research hotspot in understanding T2DM pathogenesis (2). In the regulatory circuitry governing lipid metabolic reprogramming, the dynamic interplay among mitochondria, the endoplasmic reticulum (ER), and lipid droplets (LDs) plays an irreplaceable central role. These three organelles establish a functionally integrated network through membrane contact sites, thereby orchestrating calcium homeostasis, lipid synthesis and trafficking, LD renewal, energy metabolism, oxidative stress, and the unfolded protein response to preserve cellular lipid homeostasis (3, 4). Under pathological conditions of T2DM, the mito-ER-LD interaction network undergoes marked remodeling, characterized by mitochondria-associated ER membranes (MAMs) disruption, altered distribution and functional balance of cytoplasmic mitochondria and peridroplet mitochondria, and abnormal LD accumulation, which in turn impairs fatty acid (FA) oxidation, promotes the accumulation of lipotoxic metabolites, and exacerbates oxidative stress and ER stress, thereby driving the onset and progression of T2DM. Accordingly, the mito-ER-LD interaction axis, as a central hub connecting lipid metabolic reprogramming, energy homeostasis, and stress responses, provides a new conceptual framework for unraveling the complex pathological network of T2DM and for identifying novel intervention targets by emphasizing inter-organelle crosstalk rather than isolated organelle function. On this basis, this review systematically summarizes the methodological approaches for detecting mito-ER-LD interactions, their structural basis, key regulatory molecules, and their mechanistic roles in lipid metabolic reprogramming in T2DM while also summarizing the effects of clinical drugs and lifestyle interventions on these interactions, with the goal of informing a deeper understanding of T2DM pathogenesis and the development of novel therapeutic strategies targeting inter-organelle crosstalk.

## Methods for Detecting Mito-ER-LD Interactions and Identifying Related Proteins

### Omics technologies

#### Lipidomics

Lipidomics is a discipline devoted to the systematic analysis of lipids and their interacting molecules in organisms, tissues, or cells, and biomolecular mass spectrometry is currently the core tool for this field. With technological advances, studies of mito-ER-LD interactions have gradually progressed from compositional analysis to dynamic characterization. Veyrat-Durebex *et al.* performed lipid analysis of MAM-enriched fractions using high-resolution mass spectrometry combined with positive- and negative-ion electrospray ionization modes, thereby revealing their distinct lipid composition and the distribution patterns of key phospholipids (5). Using dynamic lipidomics combined with stable isotope labeling and high-resolution mass spectrometry analysis, Renne *et al.* found that lipid transport displays molecular species selectivity and is driven by lipid concentration gradients, while membrane contact sites promote lipid movement and are crucial for maintaining mitochondrial lipid homeostasis (6). Sassano *et al.* combined liquid chromatography-tandem mass spectrometry (LC-MS)-based oxidized lipidomics with super-resolution live-cell imaging to analyze, identify, and quantify lipid peroxidation products and to map early peroxidation sites (7). Guo *et al.*, using LC-MS/MS-based targeted lipidomics, demonstrated that voltage-dependent anion-selective channel protein 3 can remodel the cellular lipid metabolic profile, providing a new omics perspective for understanding lipid signaling between organelles (8).

#### Proteomics

Proteomics is the field devoted to the study of the full complement of proteins in a specific cell type, tissue, or body fluid, including their composition, functions, and interactions. Its principal methodologies include two-dimensional gel electrophoresis, differential gel electrophoresis, and mass spectrometry. With technological advances, the detection of mito-ER-LD contact sites and their associated proteins has progressed from the early stage of only roughly isolating target proteins and preliminarily mapping proteomes to the precise identification of contact site-associated proteins and the tracking of their structural and spatial changes during specific cellular processes. Zhou *et al.* developed a novel proteomic analysis strategy termed bimolecular fluorescence complementation-proximity labeling based on bimolecular fluorescence complementation and proximity labeling, enabling highly precise enrichment of the proteomes of mito-ER contact sites (MERCs) and LD-mito contact sites in living cells (9). The orthogonal proximity labeling technique OrthoID, developed by Lee, Sung, and colleagues, combines engineered enzymes with high-affinity binding pairs and can not only identify core contact site proteins but also track protein sets that undergo structural and spatial changes during specific cellular processes, thereby revealing their dynamic behavior (10). Using CRISPR-Cas9-engineered cell lines together with proximity proteomics, immunoprecipitation, and mass spectrometry (immunoprecipitation and mass spectrometry), Nakamura *et al.* identified FK506-binding protein 8 on the outer mitochondrial membrane (OMM) as an interacting ligand of the ER protein PDZ domain-containing protein 8 (PDZD8), and these two proteins function in an interdependent manner to regulate mitochondrial morphology (11).

#### Metabolomics

Metabolomics is the field devoted to the qualitative and quantitative analysis of all low-molecular-weight metabolites in a given organism or cell, together with their dynamic detection. Currently, the main methodologies used in metabolomics include nuclear magnetic resonance (NMR), gas chromatography-mass spectrometry (GC-MS), and LC-MS, all of which offer important insights into mito-ER-LD interactions. By combining ^1^H^1^D NMR with functional experiments, Madreiter-Sokolowska *et al.* demonstrated that strengthened mito-ER contacts in senescent endothelial cells promote increased mitochondrial Ca^2+^ uptake and enhanced tricarboxylic acid (TCA) cycle activity (12). Using ^13^C_6_-glucose tracing together with LC-MS and GC-MS, Göbel *et al.* further showed that the integrity of MERCs in astrocytes does not significantly affect overall core metabolism, particularly TCA metabolism, but is essential for preserving the local metabolic microenvironment (13). Using untargeted lipidomic profiling, Maio *et al.* found that knockdown of basic leucine zipper and W2 domains 2 markedly reduced TAG levels, confirming that this protein influences lipid homeostasis by regulating MERCs (14).

#### Spatial omics

Spatial omics comprises omics-based technologies that perform in situ high-throughput profiling and analysis of biomolecules under conditions that preserve the spatial organization of tissues or cells. The major techniques include high-resolution mass spectrometry imaging approaches such as gas cluster ion beam secondary ion mass spectrometry, matrix-assisted laser desorption/ionization, and desorption electrospray ionization, which provide critical technical support for elucidating the functional mechanisms of inter-organelle interactions. Han *et al.* developed a proximity-labeling tool based on split engineered ascorbate peroxidase 2 using directed evolution, enabling spatially specific protein labeling at MERCs and providing a precise chemical tool for dissecting the dynamic proteome at organelle interaction interfaces (15). By integrating single-cell phenomics with spatial proteomics, Kang *et al.* identified spatiotemporal dynamic remodeling of LD-mito contact sites in liver lobules during starvation and demonstrated that the phosphorylation state of perilipin 5 (PLIN5) dictates LD-mito contact sites formation and function, thereby modulating lipid metabolism (16).

#### Transcriptomics

Transcriptomics is the field devoted to the global analysis of all RNA transcripts in cells, and whole-cell sequencing combined with sequencing of subcellular fractions has emerged as an important approach for elucidating the mechanisms underlying organelle interactions. Anastasia *et al.* conducted RNA sequencing on fractions enriched for rough ER-associated mitochondria and identified 205 enriched transcripts, including microsomal triglyceride transfer protein and apolipoprotein B, both of which are associated with very-low-density lipoprotein (VLDL) biogenesis, thereby providing direct evidence for the involvement of rough ER-associated mitochondria in lipoprotein synthesis (17). Li *et al.* performed RNA sequencing on renal cortical tissue and found that treatment with NSC228155 significantly downregulated tumor protein p53 and apoptotic pathways while enriching metabolism-associated pathways, suggesting that NSC228155 mitigates kidney injury through regulation of mito-ER homeostasis (18).

### Other methods

In addition to the omics technologies described earlier, various imaging and biochemical methods have also been widely applied for the direct or indirect detection of mito-ER-LD contact sites (19, 20, 21) (Table 1). These methods differ in their emphasis on resolution, dynamic analysis, or in situ characterization. For example, electron microscopy and cryo-electron tomography can provide nanometer-scale ultrastructural and three-dimensional information under near-physiological conditions, confocal microscopy is suitable for dynamic observation in living cells, and co-immunoprecipitation can identify native interacting protein complexes. These techniques each have distinct advantages and limitations, and by complementing omics-based methods, they have jointly promoted a deeper understanding of organelle interaction networks.

**Table 1:** Techniques for Investigating Mito-ER-LD Contact Sites

Techniques	Advantages	Limitations
Electron microscope	Provides ultrastructural information at nanometer-scale resolution. Enables direct visualization of contact sites between organelles.	Low throughput makes large-scale quantitative analysis difficult. It cannot monitor dynamic processes in real time.
Single-molecule tracking photoactivated localization microscopy	Offers nanometer-scale spatial resolution. Allows real-time monitoring of single-molecule movement trajectories in live cells and can capture transient events.	Limited temporal resolution. The technique is technically demanding and requires specific fluorescent protein labeling. Low-throughput and requires complex data analysis.
Confocal microscope	Three-dimensional imaging and suitable for dynamic observation in living cells.	Photobleaching and phototoxicity. Spatial resolution is limited by the diffraction barrier.
Cryo-electron tomography	Allows three-dimensional in situ imaging in a near-physiological state. No staining or fixation is required.	Low throughput and data processing is technically demanding. A relatively high technical threshold.
Light-microscopy and colocalization analysis	Captures the dynamic properties of contact sites. Overcomes the low-throughput limitation of electron microscopy.	Generally applicable only to flat, adherent cell lines. Spatial resolution is insufficient for accurate measurement of individual contact sites, which may lead to false-positive results.
Super-resolution imaging	With enhanced lateral resolution, it allows visualization of inter-organelle contact sites.	Significant photobleaching, and relatively high cost.
Co-immunoprecipitation	Captures native protein interaction complexes under near-physiological conditions and directly validate protein-protein interactions.	Unable to discriminate between direct and indirect interactions. Highly dependent on antibody specificity. Weak or transient interactions are easily lost.
Immunofluorescence microscope	High specificity. Multiple targets can be labeled simultaneously using multicolor labeling.	Requires cell fixation and permeabilization. Not suitable for dynamic tracking in live cells.
Immunohistochemistry	Enables precise in situ localization of the cellular and subcellular distribution of target proteins within tissues while preserving tissue morphology.	The results are mostly semi-quantitative. Highly dependent on antibody specificity.
Fluorescence resonance energy transfer	Highly sensitive to intermembrane distance. Suitable for live-cell applications and can be used for dynamic monitoring.	The method has demanding equipment requirements and is susceptible to artifacts.
Biochemical separation (e.g. density gradient centrifugation)	Can be used for proteomic and lipidomic profiling of contact sites, as well as for biochemical characterization.	Purity is difficult to ensure, and cross-contamination is common. Mature isolation protocols are still lacking for most contact sites.
Functional assessment (e.g. Ca^2+^ transfer/lipid synthesis)	Reflects the functional state of contact sites. The earliest reported method for assessing mitochondria-associated endoplasmic reticulum membrane function.	An indirect measurement. Multiple variables are difficult to control.

## Effects of Mito-ER-LD interactions on lipid metabolism

Recent studies have revealed that mito-ER-LD contacts are not merely structural proximity but collectively shape cellular lipid metabolic reprogramming through lipid synthesis, LD expansion, FA mobilization, mitochondrial oxidation, and lipotoxicity buffering (Fig. 1).

**Fig. 1:**
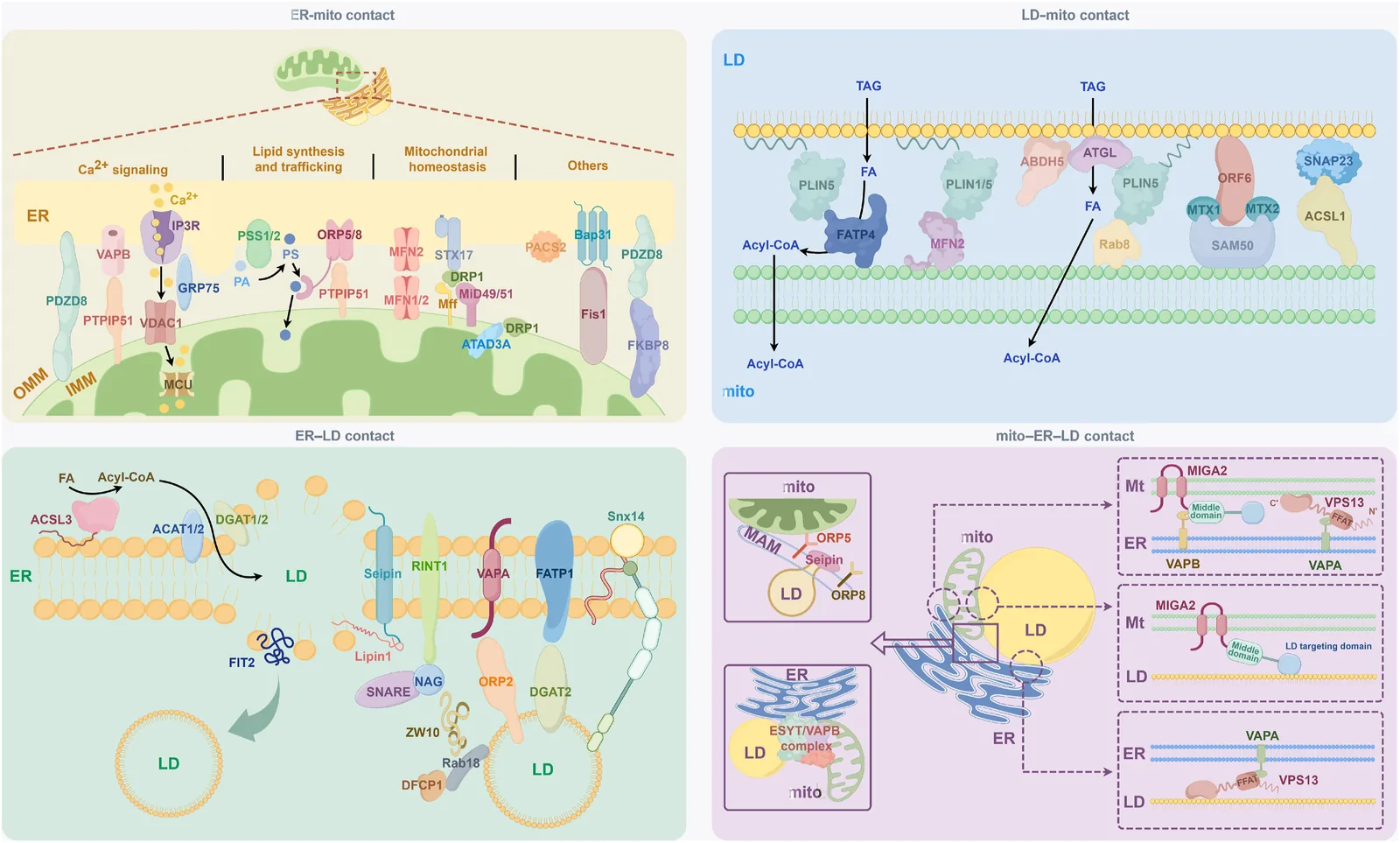
Molecular organization and functional roles of mitochondria-endoplasmic reticulum-lipid droplet contact sites. The upper-left panel summarizes mitochondria-endoplasmic reticulum contacts involved in calcium transfer, phospholipid synthesis and trafficking, mitochondrial dynamics and quality control, and other tethering functions. The upper-right panel shows lipid droplet-mitochondria contacts that couple lipolysis and fatty-acid activation to mitochondrial fatty-acid utilization. The lower-left panel depicts endoplasmic reticulum-lipid droplet contacts governing neutral-lipid synthesis, lipid droplet budding, and membrane tethering. The lower-right panel illustrates proposed tripartite mitochondria-endoplasmic reticulum-lipid droplet junctions. Abbreviations: ABDH5, alpha/beta hydrolase domain-containing protein 5; ACAT1/2, acyl-CoA:cholesterol acyltransferases 1 and 2; ACSL1/3, acyl-CoA synthetase long-chain family members 1 and 3; ATAD3A, ATPase family AAA domain-containing protein 3A; ATGL, adipose triglyceride lipase; BAP31, B-cell receptor-associated protein 31; Ca2+, calcium ion; CoA, coenzyme A; DFCP1, double FYVE-containing protein 1; DGAT1/2, diacylglycerol acyltransferases 1 and 2; DRP1, dynamin-related protein 1; ER, endoplasmic reticulum; ESYT, extended synaptotagmin; FA, fatty acid; FATP1/4, fatty acid transport proteins 1 and 4; FFAT, two phenylalanines in an acidic tract; FIT2, fat storage-inducing transmembrane protein 2; FKBP8, FK506-binding protein 8; GRP75, glucose-regulated protein 75; IMM, inner mitochondrial membrane; IP3R, inositol 1,4,5-trisphosphate receptor; LD, lipid droplet; MAM, mitochondria-associated endoplasmic reticulum membrane; MCU, mitochondrial calcium uniporter; MFN1/2, mitofusins 1 and 2; MFF, mitochondrial fission factor; MiD49/51, mitochondrial dynamics proteins of 49 and 51 kDa; MIGA2, mitoguardin 2; mito, mitochondria; MTX1/2, metaxins 1 and 2; OMM, outer mitochondrial membrane; ORF6, open reading frame 6 protein; ORP2/5/8, oxysterol-binding protein-related proteins 2, 5, and 8; PA, phosphatidic acid; PACS2, phosphofurin acidic cluster sorting protein 2; PDZD8, PDZ domain-containing protein 8; PLIN1/5, perilipins 1 and 5; PS, phosphatidylserine; PSS1/2, phosphatidylserine synthases 1 and 2; PTPIP51, protein tyrosine phosphatase-interacting protein 51; Rab8/18, Ras-related proteins Rab-8 and Rab-18; RINT1, RAD50-interacting protein 1; SAM50, sorting and assembly machinery component 50; SNAP23, synaptosome-associated protein 23; SNARE, soluble N-ethylmaleimide-sensitive factor attachment protein receptor; Snx14, sorting nexin 14; STX17, syntaxin 17; TAG, triacylglycerol; VAPA/B, vesicle-associated membrane protein-associated proteins A and B; VDAC1, voltage-dependent anion channel 1; VPS13, vacuolar protein sorting 13 homolog; ZW10, zeste white 10.

### Role of mito-ER coupling in maintaining lipid homeostasis

For decades, scientists viewed organelles as relatively independent entities. Recent research has revealed a far more interconnected and fluid cellular landscape, where organelles interact and communicate in sophisticated ways. At the heart of this paradigm shift is the discovery of specialized regions, where two critical organelles, mitochondria and ER, are in close apposition. These regions are termed MAMs, also known as MERCs (22). Indeed, as early as the 1950s, the Wilhelm Bernhard laboratory first revealed the structural characteristics of these regions using transmission electron microscopy (TEM) (23, 24). MERCs act as cellular “communication hubs,” allowing for rapid and precise exchange of signals and molecules between mitochondria and the ER. This communication is crucial for maintaining cellular homeostasis, responding to stress and regulating energy metabolism (25, 26).

#### Structure and characteristics of MERCs

##### Structure–function Coupling and Sub-specialization

One of the core features of MERCs is that their structural heterogeneity corresponds to the sub-specialization of functional modules (27). Their morphological characteristics are mainly defined by contact area, lateral extent, and intermembrane distance and are regulated by the local membrane protein composition and tethering complexes (19). Under physiological resting conditions, MERCs display an intermembrane distance of approximately 10∼30 nm, with the spacing varying according to ER subtype, at about 10 nm for smooth ER and about 25 nm for rough ER (28, 29). Based on variations in contact area, MERCs can be categorized into several types, highlighting their structural adaptability under distinct cellular and metabolic conditions (30, 31).

The functional diversity of MERCs is mirrored in their structural heterogeneity, with sub-specialization likely supported by multiple, different linker-forming protein structures. Over the years, a growing number of ER and mitochondrial proteins have been identified as components of MERCs, and their functions are being gradually revealed (32). For example, Ca^2+^ transport-related proteins include the IP3R/GRP75/VDAC1 complex, Sig1R, and PDZD8; lipid synthesis and transfer-associated proteins include PSS1/2, DGAT2, and the ORP5/ORP8-PTPIP51 complex; mitochondrial dynamic regulatory proteins include DRP1 and FUNDC1; MERCs tethers proteins include the VAPB-PTPIP51 complex, VPS13A, and BAP31; and MERCs controlled proteins, acting as promoter or disruptor, include PACS2 and MFN2 (33, 34, 35, 36, 37, 38, 39, 40, 41, 42, 43, 44, 45, 46, 47, 48, 49, 50, 51, 52, 53, 54). Table 2 summarizes detailed information on MERCs-associated proteins.

**Table 2:** Mitochondria-Endoplasmic Reticulum Contact Site-Resident Proteins

Function Types	Proteins	Organellar Localization	Relevant Interactions	Function and mechanism
Ca^2+^ transport	Inositol1,4,5-trisphosphate receptor (IP3R)	ER	Forming IP3Rs/GRP75/VDAC1 complex	Regulate the transfer of Ca^2+^ from the ER to the mitochondrial matrix via the mitochondrial calcium uniporter (MCU). The deglycase (DJ-1) can form a tetramer complex with VDAC, IP3R, and GRP75 to regulate the transfer of Ca^2+^.
	Glucose regulated protein 75 (GRP75)	mitochondria		
	voltage-dependent anion channel 1 (VDAC1)	OMM		
	Sigma-1 receptor (Sig1R)	ER	Protects IP3R from ER-associated (protein) degradation	Promoting the stabilisation of IP3R, prolonging Ca^2+^ signalling into the mitochondria.
	PDZD8	ER	Forming PDZD8-FKBP8 complex	PDZD8 is necessary for the formation of ER-mito contacts in mammalian cells, and is required for efficient Ca^2+^ transfer from ER into mitochondria. It contains a synaptotagmin-like mitochondrial-lipid-binding protein domain, predisposing it to mediate lipid transfer at MERCs. PDZD8-FKBP8 complex plays an essential role in MERCs formation and the regulation of mitochondrial morphology.
Lipid metabolism	Phosphatidylserine synthase 1 and 2 (PSS1/2)	mitochondria-associated ER (ER_mt_)	/	Synthetic phosphatidylserine (PS).
	Diacylglycerol O-acyltransferase 2 (DGAT2)	ER	Interacting tightly with mitochondria	Catalyzing the final step of triacylglycerols (TAG) synthesis.
	Oxysterol-binding protein-related protein five-eighths (ORP5/8)	ER, ER_mt_	Binds PTPIP51	Mediating the transfer of PS from ER to mitochondria.
	Protein tyrosine phosphatase-interacting protein 51 (PTPIP51)	OMM	Binds VAPB, ORP5/8	
Mitochondrial dynamics	Dynamin-related protein 1 (DRP1)	OMM	Interacts with SPIRElc, INF2, ATAD3A	Regulating mitochondrial fission.
	FUN14 domain-containing protein 1 (FUNDC1)	OMM	Binds IP3R	Mitophagy receptor.
MERCs tethers proteins	Vesicle-associated membrane protein B (VAPB)	ER, ER_mt_	Binds PTPIP51	The VAPB-PTPIP51 interaction regulates ER-mito associations. Recent research has underscored the crucial role of the VAPB-PTPIP51 tether in facilitating key processes such as ER-mitochondrial Ca^2+^ exchange, autophagy, synaptic activity, synthesis, and transport of lipids.
	Vacuolar protein sorting 13A (VPS13A)	ER_mt_	Interacting with the ER protein VAPA	Promoting MERCs formation and mitochondria stability. Involved in mitophagy.
	B-cell receptor-association protein 31 (BAP31)	ER	Interacts with FIS1, TOMM40	The BAP31 and FIS1 tether pair connecting mitochondria and ER and were found to be involved in apoptotic signaling by activating procaspase 8. BAP31 is a key factor in maintaining the integrity of MERCs with translocase of the outer mitochondrial membrane 40 (TOMM40), and the BAP31-TOMM40 complex is essential for activating mitochondrial respiration by stimulating NDUFS4 translocation in mitochondria by regulating the activity of the mitochondrial respiratory chain complex I.
MERCs controlled proteins	phosphofurin acidic cluster sorting protein 2 (PACS2)	ER, mitochondria	Stabilizes BAP31	Required for MERCs stabilization; Regulation of Ca^2+^ transfer and phospholipid synthesis; Inhibiting apoptosis. Loss of PACS2 results in BAP31-mediated fragmentation of the mitochondria, depletion of Ca^2+^ signal and a decrease in MERCs itself.
	Mitofusin-2 (MFN2)	OMM, ER	Interacting with MFN1/2 to form homodimeric or heterodimeric	MFN2 is one of the most well-studied, yet most controversial, MERCs tethers. MFN2 is known for its role in mitochondrial fusion, but also localizes to the ER and thus can link the ER with the OMM. However, lately it has been under intense debate whether MFN2 promotes or hinders ER-mito contact formation owing to seemingly conflicting outcomes of over-expression and silencing experiments. Overall, it is accepted that MFN2 is a key component of MERCs and is important in the appropriate functioning of MERCs.

##### High Dynamicity and tissue Heterogeneity

MERCs are highly dynamic rather than static structures and can exhibit plastic alterations in morphology and composition in response to cellular stress and metabolic requirements. Structural dynamics of MERCs can precisely modulate the cellular pathways they mediate, thereby affecting related functions (55). This dynamicity is mediated by the rapid association and dissociation of tethering proteins, as well as by the local or distal actions of regulatory factors. Meanwhile, the protein composition of MERCs exhibits marked organ- and tissue-specific differences. According to previously published mass spectrometry analyses, proteins enriched at MERCs vary markedly, depending on the organ from which they had been extracted. This tissue specificity is further highlighted by the fact that mutated forms of ubiquitously expressed MERCs proteins result in organ-specific pathologies (56, 57).

#### Functions of mito-ER contacts

##### Lipid Synthesis and nonvesicular transport

The role of MERCs in maintaining lipid homeostasis has been firmly demonstrated, as they create a hydrophilic interface between the ER and mitochondria that supports bidirectional nonvesicular lipid transfer and concentrates key enzymes and complexes required for lipid synthesis and transport (58). The first described function of MERCs was exchange of phospholipid (PL) between organelles (59). PLs shuttle between the ER and mitochondria. Phosphatidic acid (PA) generated in the ER is converted into PS by PSS1/2 at MERCs and subsequently imported to mitochondria via ORP5/ORP8-PTPIP51 interactions. The imported PS is decarboxylated into phosphatidylethanolamine (PE) by PtdSer decarboxylase 1 in the inner mitochondrial membrane. Subsequently, PE returns to the ER and methylates via PEMT2 at MERCs to generate phosphatidylcholine (60, 61). Recent studies have shown that the VAPB-PTPIP51 complex regulates the transfer of PA in MERCs (62). MFN2 could selectively bind to PS and transfer it to mitochondria. Knockout of MFN2 reduced the expression of PSS1 and decreased the transfer of PS from the ER to the mitochondria, resulting in impaired phospholipid synthesis and ER stress (63). In addition to phospholipid metabolism, MERCs are also enriched with key proteins such as DGAT2, StAR, VDAC2, CAV1, FACL4, and SOAT1 (also known as ACAT1), which participate in neutral lipid synthesis, steroidogenesis, and LD formation. Thus, MERCs function not only as conduits for lipid exchange but also as important subcellular platforms that regulate lipotoxicity and energy homeostasis.

##### Ca^2+^ Signaling

MERCs are central hubs for cellular Ca^2+^ signaling, providing a high concentration of Ca^2+^ microregions conducive to efficient mitochondrial Ca^2+^ uptake and serving as key structures for maintaining Ca^2+^ homeostasis between the ER and mitochondria. The ERMCs are formed between IP3Rs on the ER membrane which tether to voltage-dependent anion channel (VDAC1) on the OMM through the chaperone glucose-regulated protein 75 (GRP75) (64). The resulting VDAC1-Grp75-IP3R complex serves as the major platform and chaperone complexes that coordinate calcium transfer between ER and mitochondria, enabling Ca^2+^ released from the ER to efficiently enter mitochondria and subsequently the mitochondrial matrix via MCU (65, 66). This process establishes local high-Ca^2+^ microregions at MERCs that are critical for sustaining mitochondrial metabolic enzyme activity, ATP generation, and oxygen consumption. The efficiency of Ca^2+^ transport depends not only on IP3R activity but is also regulated by Sig1R, SERCA, and luminal Ca^2+^-binding proteins such as CNX and BiP/glucose-regulated protein 78 (GRP78) in MERCs (35, 67, 68, 69, 70, 71, 72, 73). Appropriate ER-to-mitochondria Ca^2+^ transfer supports mitochondrial metabolism, whereas persistent Ca^2+^ overload may cause mitochondrial dysfunction, enhance DRP1-dependent mitochondrial fission, and induce mPTP opening together with cytochrome c release, ultimately resulting in cell injury or even apoptosis (74, 75, 76).

##### Mitochondrial Homeostasis

MERCs have a key role in maintaining efficient mitochondria quality control mechanisms and mitochondrial health. In mitochondrial dynamics, MERCs are regarded as one of the primary initiation sites of mitochondrial fission. DRP1 can be recruited and assembled at MERCs, where it cooperates with fission proteins such as Fis1, Mff, and MiD49/51 to initiate membrane constriction and fission (77). Several other MERCs-associated proteins, such as INF2, FUNDC1, PACS2, and STX17, also participate in mitochondrial fission (78, 79, 80, 81). In addition to participating in mitochondrial fission, MERCs are also closely associated with mitochondrial fusion and intracellular trafficking. For example, MFN2 is involved in mitochondrial fusion, whereas the MIRO complex regulates mitochondrial motility (82, 83, 84). In mitochondrial quality control, multiple autophagy-related proteins relocalize to MERCs under stress or starvation conditions, thereby promoting autophagosome membrane formation and the clearance of damaged mitochondria. The PINK1/Parkin pathway, together with BECN1, FUNDC1, and STX17, has been implicated in MERCs-dependent mitophagy (85, 86).

##### ER Stress and unfolded protein response (UPR)

MERCs are closely associated with ER stress and the UPR. As a central organelle for protein folding, lipid synthesis, and Ca^2+^ storage, the ER activates the UPR in response to the accumulation of misfolded proteins, primarily through the protein kinase R-like endoplasmic reticulum kinase (PERK), inositol-requiring enzyme 1 alpha (IRE1α), and activating transcription factor 6 (ATF6) pathways (87). Moderate activation of the UPR can alleviate stress by upregulating the expression of chaperone proteins such as BiP/GRP78, suppressing protein translation, and promoting mitochondrial ATP production. However, persistent or excessive stress can aggravate Ca^2+^ imbalance and mitochondrial damage through MERCs, thereby inducing apoptosis (88, 89). Studies have demonstrated that both PERK and IRE1α are localized at MERCs. Interaction between PERK and MFN2 is essential for UPR activation and contributes to the functional regulation of MERCs while also modulating reactive oxygen species (ROS) responses (90, 91). Under ER stress conditions, IRE1α is regulated by OMM-associated ubiquitination mechanisms, thereby affecting its oligomerization and pro-apoptotic signaling output (92, 93). Moreover, during ER stress, ATF6 can promote MERCs formation and enhance Ca^2+^ transfer from the ER to mitochondria (94). The above evidence suggests that MERCs are not only response sites for ER stress signaling but also important interfaces through which stress is transmitted to mitochondria and cell fate is determined.

##### Autophagy and Apoptosis

MERCs serve as integrative platforms in cell fate decisions, including autophagy, apoptosis, and necroptosis. On the one hand, MERCs have been demonstrated to mediate autophagosome formation in mammalian cells. Under starvation conditions, autophagy-related proteins, including ATG14, ATG6, Beclin-1, STX17, and ZFYVE1/DFCP1, are recruited to MERCs, thereby promoting autophagosome formation (86, 95). PTPIP51 has been reported to bind VAPB and regulate autophagy. Increasing MERCs formation by overexpressing VAPB or PTPIP51 impairs autophagosome formation (96). On the other hand, MERCs directly contribute to apoptotic signaling cascades through modulation of Ca^2+^ homeostasis and ROS signaling (97, 98). Numerous proteins are involved in the regulation of the calcium transfer axis in MERCs, thereby precisely regulating cell survival and death. For example, c-FLIPL inhibits Ca^2+^ transport at MERCs and exerts anti-apoptotic effects, whereas Bcl-xl, Bcl2l10, and IRBIT participate in apoptotic regulation by modulating IP3R-mediated Ca^2+^ release (99, 100, 101). Notably, MERCs also participate in the regulation of necroptosis. Components of the RIP1/RIP3/MLKL complex associated with necroptosis are all localized at MERCs (102, 103).

In summary, MERCs play a pivotal role, not only in maintaining the functional integrity of the cellular environment but also in responding to metabolic cues and stress signals. Structural and functional disruption of MERCs can directly influence lipid synthesis and trafficking and can also promote insulin resistance and impair lipid metabolism through the induction of mitochondrial dysfunction, Ca^2+^ homeostatic imbalance, and enhanced oxidative stress, thereby resulting in lipid accumulation and lipotoxicity (104, 105, 106).

### Role of ER-LD crosstalk in maintaining lipid homeostasis

LDs are dynamic organelles in eukaryotic cells, comprising phospholipid monolayers surrounding a central core containing predominantly TAG and sterol ester (SE) (107). Because of their unique structure, LDs’ surface is targeted by peripheral proteins, which are key to most LD functions. Many of these proteins are cytosolic (class II) and localize to LDs through the CYTOLD pathway (for cytosol-to-LD) via amphipathic helices, lipid anchors, or adaptor proteins. Other proteins (class I) move to the LD from the ER using the ERTOLD pathway (for ER-to-LD), typically having monotopic domains that allow diffusion between the ER and LD surfaces through their physical continuity (108).

LDs are considered cellular energy storage organelles. Besides the energy storage function, increasing evidence shows that LDs are highly dynamic, alternating between periods of growth and consumption by lipolysis or lipophagy. Another, and perhaps less appreciated, role of LDs is in the protection against lipotoxicity by sequestering FAs. Accordingly, conditions in which FA storage in LDs is impaired or the storage capacity is overwhelmed can result in lipotoxicity. LDs originate from the ER. Despite the possibility to detach, LDs remain in contact with the ER. Furthermore, LDs are reported to actively communicate with other cellular organelles like endosomes/lysosomes and mitochondria (109). Contact sites of LD with the ER supply to the LD the FAs that are stored in their core as neutral lipids. These FAs can be transferred to mitochondria to be used for energy production by β-oxidation. Also, during LD biogenesis and growth, specific lipids (i.e. PLs) should be transported from the ER to the growing LD to regulate their size and to maintain a balanced lipid monolayer composition.

#### LD biogenesis in the ER

The biogenesis of LDs involves the deposition of neutral lipids between the leaflets of the ER, the phase separation of neutral lipids into a lens-like structure, and the emergence of the LD from the outer leaflet of the ER into the cytoplasm (110). The first step in LD biogenesis is the synthesis of neutral lipids, most commonly TAGs and SEs. These result from the esterification of an activated FA to diacylglycerol (DAG) or a sterol (such as cholesterol), respectively. Different enzymes are involved in each case, all localized primarily to the ER. SEs are made by acyl-CoA:cholesterol O-acyltransferase (ACAT1 and ACAT2), whereas triacylglycerols are the product of DAG acyltrans-ferases (DGAT1 and DGAT2) (111). In this process, Acyl-CoAs made by acyl-CoA synthetase are required in cholesterol esterification processes. In particular, ACSL3, a key acyltransferase in the generation of TAG in mammalian cells, is related to LD formation (112). Synthesized neutral lipids are accumulated between the leaflets of the ER bilayer, where they form a lens-like structure (a nascent LD). Subsequently, the coalescence of neutral lipids buds out towards the cytosol and eventually becomes independent from the ER (113).

Other candidate proteins are also related to LD formation, including PLIN family members, FIT, LDAF1, and Seipin. PLINs may aid in LD budding by accumulating at ER regions containing high contents of neutral lipids or DAG and may thus help to stabilize nascent forming LDs (114). In cells, lipid droplet budding is facilitated by fat storage-inducing transmembrane (FIT) proteins. However, how FIT proteins promote LD budding remains unclear. Earlier studies showed that FIT2 could promote LD budding through direct binding to DAGs and TAGs in vitro (115). Seipin is a widely conserved ER membrane protein required for correct LD budding. In the absence of seipin, LD formation is delayed and there are lower incorporation rates of both proteins and lipids into the LDs that form, resulting in droplets with aberrant morphologies and properties (116, 117). In human cells, LDAF1 is required for normal LD formation. The structure of LDAF1 is unknown but it interacts with the hydrophobic helix of seipin and localizes to the sites of LD formation. The function of LDAF1 is unclear but LDs form abnormally in cells lacking LDAF1. There are fewer and larger LDs, and they form at an apparently higher cellular TAG concentration (118).

Spatial definition of LD biogenesis, however, does not only depend on specific proteins but also requires local enrichment of particular lipids, whose biochemical and biophysical properties promote the assembly of LD biogenesis factors. Lipin1, which generally localizes in the cytoplasm, also functions as a regulator of TAG synthesis via the generation of DAG (119). Interestingly, it has been observed that lipin1 interacts with seipin (120, 121). Based on theoretical and experimental evidence, the formation of ER-LD junctions is affected by the intrinsic molecular curvature of ER phospholipids. While lipids that promote negative curvature such as DAG stabilize an ER embedded state of LDs, lipids that promote positive membrane curvature, such as lysophospholipids, will favor emergence of LDs toward the cytoplasm. In addition, the directionality of LD emergence from the ER, that is, whether LDs bud toward cytoplasm or the ER lumen, is affected by an asymmetric distribution and packaging of lipids at LD biogenesis sites, surface tension, membrane curvature properties, and lateral pressure. Even the acyl composition on ER phospholipids affects LD formation as lipids containing short chain and saturated FAs antagonize LD formation and instead promote neutral lipid accumulation in the ER (122).

#### Structure of ER-LD contacts

The ER is not only the site of LD biogenesis but may be a life-long partner of this organelle. Once formed, LDs keep close contact to the mother organelle and that this may be achieved via several, morphologically distinct and potentially functionally specialized connections, enabling dynamic remodeling of LDs in response to cellular energy status and needs (123, 124, 125, 126, 127, 128, 129, 130, 131, 132, 133, 134, 135, 136, 137) (Table 3). LD biogenesis and degradation, as well as their interactions with ER, are tightly coupled to cellular metabolism and are critical to buffer the levels of toxic lipid species.

**Table 3:** Endoplasmic Reticulum-Lipid Droplet Contact Proteins

Name	Proposed Function at ER-LD Contacts
Seipin	Seipin is stably localized to ER-LD contact sites and regulates the contacts of newly formed LDs with the ER, facilitating the transfer of protein and lipid cargo between them. Deletion of BSCL2 (encoding seipin) in human cells disrupted the morphology of ER-LD contacts and some LDs completely detached from the ER.
LD organization (LDO) and promethin/LD assembly factor 1 (LDAF1)	The LDO proteins, LDO16, and its splice variant LDO45 were recently identified as genetic interactors of seipin. LDO16 and LDO45 play important roles in regulating LD biogenesis at a spatially confined ER domain adjacent to the nuclear-vacuolar junction (NVJ), a large contact site between the nuclear ER and the vacuole, during nutritional stress. LDO45 promotes TAG accumulation and hence proliferation of LDs, whereas LDO16 is necessary for efficient lipophagy, LD consumption by autophagy. Promethin/LDAF1 is a remote homolog of LDO45 and a seipin partner protein identified in humans. In mammalian cells, seipin collaborates with promethin/LDAF1 to define discrete ER sites of LD biogenesis.
FIT2	Shuffling TAG to forming LDs, decreasing DAG at LD-forming sites to allow for LD emergence, regulating LD-budding to cytosol.
Rab18	Rab18 participates in mediating the formation of ER-LD contacts. Overexpression of Rab18 greatly enhances ER-LD junctions possibly by decreasing PLIN2 levels on LDs. Rab18 interacts with the ER-associated NBAS-RINT1-ZW10 tethering complex and their associated SNARES, resulting in the recruitment of ER to LDs and the formation of direct ER-LD junctions thereby promoting lipogenesis and LD growth. Another partner protein of Rab18 is the double zinc finger FYVE domain-containing protein 1 (DFCP1), lack of which leads to decreased ER-LD junctions, whereas overexpression of DFCP1 results in increased junction formation.
DGAT2-FA transport protein 1 (FATP1)	The FATP1 is an ER protein while DGAT2 is present in LDs, and the interaction between them is thought to be linked to TAG synthesis and its incorporation into LDs.
Oxysterol-binding protein-related protein 2 (ORP2)-vesicle-associated membrane protein-associated protein A (VAPA)	Overexpression of ORP2 and VAPA facilitates their interaction at adjacent clustered LDs, which induced increases in LDs and TAG synthesis.
Vacuole membrane protein 1 (VMP1)	May modulate sarcoplasmic/ER calcium ATPase (SERCA) activity, knockdown increases LD proximity to ER.
Interactor of little elongation complex ELL subunit 2 (ICE2)	Channelling DAG from LDs to ER.
Motile sperm domain-containing protein 2 (MOSPD2)	MOSPD2 forms ER-LD contacts, thanks to its CRAL-TRIO domain. Remarkably, the absence of MOSPD2 markedly disturbs the assembly of LDs.
Snx14	Snx14 acts as an ER-LD tether and promotes LD biogenesis.

### Role of LD-mito contacts in maintaining lipid homeostasis

Multispectral time-lapse imaging analysis has revealed that mitochondria are the second most common interaction partners of LDs after the ER (138). LD-mito contacts contribute to lipid homeostasis by coordinating FA storage and utilization in response to cellular metabolic demands. (139).

#### Mitochondrial heterogeneity in LD-mito interactions: Cytoplasmic mitochondria and peridroplet mitochondria

LD-mito contact can be divided into dynamic “kiss-and-run” model and stable anchoring (140, 141, 142). This classification represents modes of contact. By contrast, current research identifies two types of mitochondria based on their contact with lipid droplets: peridroplet mitochondria and cytoplasmic mitochondria. Peridroplet mitochondria are physically attached to LD, while cytoplasmic mitochondria are spatially separated from LD. Peridroplet mitochondria have distinct bioenergetics, proteome, cristae organization, and dynamics compared to cytoplasmic mitochondria (143). In brown and beige adipocytes, peridroplet mitochondria display enhanced pyruvate oxidation, electron transport and ATP-synthetic capacity, while cytoplasmic mitochondria have increased FA oxidation and uncoupling capacities. The ATP generated by peridroplet mitochondria supports local FA activation, triacylglycerol synthesis and LD expansion. Furthermore, peridroplet mitochondria maintain a distinct protein composition due to uniquely low fusion-fission dynamics (144, 145, 146). In hepatocytes, FAs are selectively trafficked to and oxidized in cytoplasmic mitochondria during fasting. However, peridroplet mitochondria facilitate FA esterification and LD expansion in nutrient-replete medium (147). Thus, LD-mito contacts could serve as sites for both lipogenesis and lipolysis in different cell types or under different metabolic conditions, rather than acting as a uniform conduit for FA delivery (Fig. 2).

**Fig. 2:**
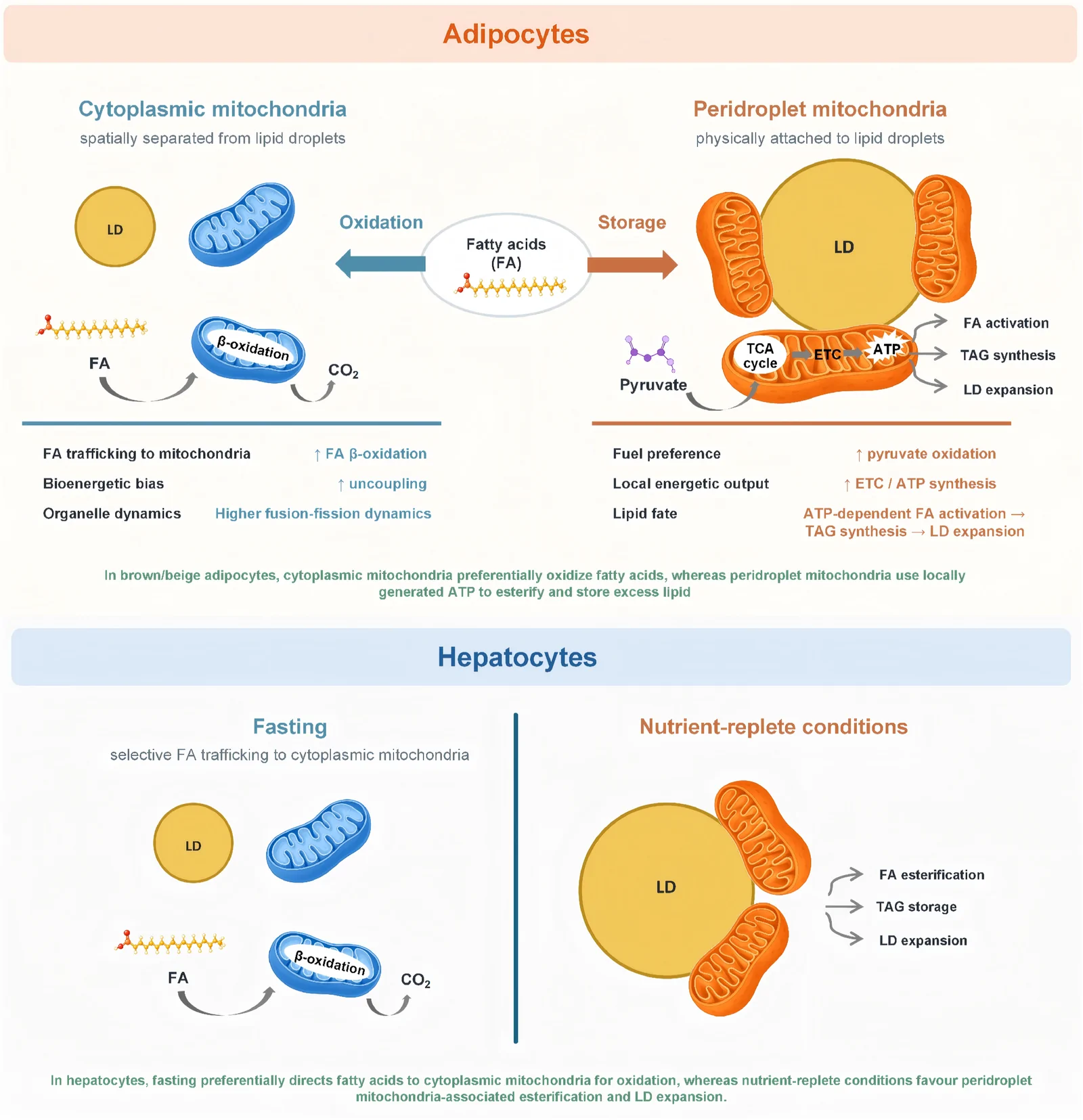
Tissue- and nutrient-state-dependent specialization of cytoplasmic and peridroplet mitochondria. In brown and beige adipocytes, cytoplasmic mitochondria are spatially separated from lipid droplets and preferentially support fatty-acid beta-oxidation and uncoupling, whereas peridroplet mitochondria are physically attached to lipid droplets and preferentially oxidize pyruvate. The resulting local adenosine triphosphate production supports fatty-acid activation, triacylglycerol synthesis, and lipid droplet expansion. In hepatocytes, fasting preferentially directs fatty acids to cytoplasmic mitochondria for oxidation, whereas nutrient-replete conditions favor peridroplet-mitochondria-associated fatty-acid esterification, triacylglycerol storage, and lipid droplet expansion. Abbreviations: ATP, adenosine triphosphate; CM, cytoplasmic mitochondria; CO_2_, carbon dioxide; ETC, electron transport chain; FA, fatty acid; LD, lipid droplet; PDM, peridroplet mitochondria; TAG, triacylglycerol; TCA, tricarboxylic acid.

The structural basis of LD-mito contacts depends on protein tethers. Several key players that are known to mediating LD-mito contact include the synaptosome-associated protein 23, mitofusin 2 (MFN2), as well as PLIN5 (139, 148, 149, 150, 151, 152, 153, 154, 155, 156, 157, 158, 159, 160, 161, 162, 163, 164, 165, 166, 167, 168) (Table 4). Tethering is not merely a structural event but can influence mitochondrial motility, fusion, substrate accessibility, and the molecular composition of LD-mito contact sites. Because LD association can restrict mitochondrial motility and fusion, changes in tethering strength or tethering proteins may reshape the relative abundance and functional state of peridroplet mitochondria and cytoplasmic mitochondria. Such remodeling can, in turn, influence the partitioning of FAs between esterification and storage in LDs and mitochondrial oxidation, thereby contributing to metabolic flexibility.

**Table 4:** Lipid Droplet-Mitochondria Contact Proteins

Proteins	Principal Localization or Features	Role in LD-Mito Interactions	Key mechanisms
PLIN5	A member of the PLIN family, predominantly expressed in highly oxidative tissues such as the heart, skeletal muscle, and brown adipose tissue.	PLIN5 sits at the LD-mito interface and is necessary and sufficient to enhance these contacts. Its loss reduces FA oxidation and TAG synthesis, while its overexpression recruits LDs to mitochondria.	It exerts dual functions, serving either as a barrier to lipolysis that favors lipid storage or as a promoter of lipolysis and FA oxidation, and this functional transition is closely linked to the phosphorylation state of PLIN5. Under energy stress or starvation, protein kinase A (PKA) catalyzes phosphorylation of PLIN5 at Ser155, thereby enhancing its interaction with FA transport protein 4 (FATP4) and MFN2 to promote TAG lipolysis and mitochondrial β-oxidation. In skeletal muscle, PLIN5 can also regulate mitochondrial FA uptake through interactions with adipose triglyceride lipase (ATGL)/abhydrolase domain containing 5 (ABDH5) and Rab8a.
SNAP23	A SNARE protein that is highly expressed in human skeletal muscle and mediates the docking and fusion of insulin-stimulated glucose transporter type 4 (GLUT4) with the plasma membrane.	SNAP23 is localized to LDs and may be present near the LD-mito contact site, thereby promoting their physical interaction and functional coupling.	SNAP23 does not directly mediate the interaction between LDs and mitochondria. Instead, it indirectly regulates the contact efficiency between LDs and mitochondria by promoting the fusion of LDs. Recent studies have further revealed that SNAP23, together with synaptic fusion protein 18 (STX18) and SEC22B, assembles to form a stable SNARE complex, which directly binds to CIDE C (FSP27) and plays a crucial role in driving lipid-content mixing during LD fusion. Under fasting conditions, SNAP23 is recruited to LDs and its expression is increased, thereby promoting physical contacts between LDs and mitochondria to maintain systemic energy homeostasis. Moreover, through interactions with vesicle-associated membrane protein 4 (VAMP4) and acyl-CoA synthetase long-chain family member 1 (ACSL1), it facilitates the anchoring of LDs to the OMM for FA oxidation.
MFN2	A classical mitochondrial fusion protein that can also participate in the formation of membrane contact sites.	MFN2 anchors mitochondria to LDs, thereby promoting the transfer and oxidation of FAs from LDs to mitochondria.	mitochondria-localized MFN2 interacts with LD-localized Hsc70 to mediate LD-mito contacts. Prolonged lipid overload induces acetylation of MFN2 at the K243 site and its subsequent degradation via the ubiquitin-proteasome pathway, thereby suppressing mitochondria-lipid droplet contact formation and leading to lipid accumulation. The coexpression of PLIN1 and MFN2 promotes lipolysis and stimulates fat oxidation, and their interaction is enhanced after stimulation by adrenaline. In contrast, the combination of MFN2 and PLIN2 is regulated by microRNA epigenetic factors and LD-mito contact and affects the reprogramming of hepatic lipid metabolism.
Vacuolar protein sorting 13 homolog D (VPS13D)	Novel regulators of LD-mito interactions.	Promote the transfer of FAs from LDs to mitochondria.	VPS13D interacts with the endosomal sorting complex required for transport I (ESCRT-I) subunit tumor susceptibility gene 101 protein (TSG101) at contact sites to remodel LDs and create a protrusion of the LD monolayer towards mitochondria.
Rab32	A GTPase localized at membrane contact sites.	Regulate LD-mito contacts and lipid metabolism-related processes.	Under high-fat diet, the GTPase Rab32 seems to be involved in TAGs synthesis and accumulation in LDs as well as the expression of genes involved in lipolysis and FA β-oxidation.
Open reading frame 6 protein (ORF6)	Coronavirus-related protein	Promote the tethering of mitochondria to LDs.	ORF6 interacts with the sorting and assembly machinery (SAM) complex in the OMM to link mitochondria to LDs. In doing so, ORF6 promotes cellular lipolysis and LD biogenesis.
Cav-1	Membrane-associated proteins	Mediate LD-mito contact.	
Ayr1	Lipid metabolic enzyme	Promote the formation of LD-mito contact sites.	The lipid metabolism enzyme Ayr1 captures the mitochondrial import complex, promoting the formation of LD-mito contact sites.
DRP1-PLIN2 axis	Mitochondrial fission-related regulatory axis	Emerging evidence suggests that excessive mitochondrial fission disrupt LD-mito contact in hepatocytes via the DRP1-PLIN2 axis.The mitochondrial fission inhibitor Mdivi-1 promoted LD-mito contact by upregulating PLIN2 and PLIN5 expression, thereby alleviating cardiac lipotoxicity.	

#### Functions of LD-mito contacts

LD-mito contacts switch between anabolic (LD expansion) and catabolic (FA oxidation) modes, depending on nutrient status and activation of signaling pathways. LD-mito contacts support bidirectional FA flux. FAs are mobilized from LDs for mitochondrial β-oxidation under energy demand and are re-esterified into TAG stored in LDs to shield mitochondria from lipotoxicity in other contexts (169, 170, 171). Moreover, the interplay between LDs and mitochondria is crucial in redox signaling. LD-mito contacts indirectly regulate ROS generation by the mitochondrial electron transport chain through modulating the rate of FA oxidation. Furthermore, LDs can sequester lipid peroxidation products, reducing the burden of oxidative stress on cells (172). Accordingly, dysregulation of the peridroplet mitochondria-to-cytoplasmic mitochondria ratio or of the mechanisms controlling this balance may contribute to oxidative stress, mitochondrial dysfunction and insulin resistance (173, 174).

### Role of mito-ER-LD crosstalk in maintaining lipid homeostasis

LDs have long been considered as inert particles used by the cells to store FAs and sterols as esterified non-toxic lipid species (i.e. TAGs and SEs) (175). The ER serves as the origin of neutral lipid synthesis and LD budding. Mitochondria provide a platform for FA β-oxidation, the TCA cycle, and ATP production. With the advancement of the technologies to study contact sites, it has become evident that the complexity of the structure and functions of contact sites go far beyond the transfer of molecules between two organelles. Multiple layers of evidence emerging from recent studies reveal that contact sites can be established simultaneously between three or even more organelles (such as mito-ER-LD) (176). mitochondria, ER, and LDs form a tightly integrated metabolic network through dynamic membrane contact sites, the core of which lies in achieving a dynamic balance among cellular energy storage (LDs), energy production (mitochondria), and lipid supply (ER) through lipid synthesis, transport, and utilization, thereby ensuring cell survival and normal physiological function under different nutritional and stress conditions.

Recently identified tri-organelle junctions linking mitochondria, ER, and LDs play an important role in coordinating LD biogenesis, directed FA transport, mitochondrial energy metabolism, and lipotoxic responses (177, 178, 179, 180, 181, 182, 183, 184, 185, 186, 187) (Table 5).

**Table 5:** Mitochondria-Endoplasmic Reticulum-Lipid Droplet Contact Proteins

Name	Localization and Characteristics	Principal Functions and Underlying mechanisms
Mitoguardin 2 (MIGA2)	MIGA2 protein localizes to ER-mito or mito-LD contact sites. An N-terminal transmembrane domain mediates its anchoring to the OMM; a middle region containing a coiled-coil domain and two phenylalanines in an acidic tract (FFAT) motif binds the ER-anchored VAPA/B proteins to promote the formation of ER-mito contact sites; and a C-terminal domain is reported to promote contacts to LDs. The crystal structures of the MIGA2 LDtargeting domain and the ER membrane protein VAPB bound to the phosphorylated FFAT motif of MIGA2 reveal that MIGA2 LD targeting domain has a large internal hydrophobic pocket that accommodates phospholipids and that two phosphorylations of the FFAT motif are required for tight interaction of MIGA2 with VAPB, which enhances the rate of lipid transport.	MIGA2 links reactions of de novo lipogenesis in mitochondria to TAG production in the ER, thereby facilitating efficient lipid storage in LDs Further biochemical studies show that MIGA2 transports phospholipids between membranes with a strong preference for binding and trafficking phosphatidylserine (PS).
Extended synaptotagmin 1/extended synaptotagmin 2 (ESYT1/ESYT2)-vesicle-associated membrane protein-associated protein B (VAPB) complex	It localizes to the mito-ER-LD interface.	It likely transfers FAs from LDs to mitochondria, thereby regulating efficient FA oxidation and the maintenance of cellular lipid homeostasis. Deletion of ESYT1, ESYT2 or VAPB limits LD-derived FA oxidation, resulting in depletion of TCA cycle metabolites, remodeling of the cellular lipidome, and induction of lipotoxic stress.
DGAT2	DGAT2 is the major TAG biosynthetic enzyme in eukaryotes. DGAT2 expressed in cultured cells localizes to the ER under basal conditions. After providing oleate to drive TAG synthesis, DGAT2 also localized to near the surface of LDs, where it co-localized with mitochondria.	DGAT2 is dynamically localized to different subcellular compartments, including the ER, LDs, and MAM/mitochondria. This localization may promote the efficient synthesis and storage of TAG in LDs.
Seipin	It is localized at ER-LD contact sites and ER-mito contact sites.	Seipin regulates LD biogenesis and maintains ER-LD contact sites. Seipin is enriched at MAMs in human and mouse cells and localizes in the vicinity of calcium regulators sarco/ER Ca^2+^-ATPase 2 (SERCA2), IP3R, and VDAC, thereby controlling mitochondrial energy metabolism by regulating mitochondrial calcium influx at MAMs.
ORP5/8	ORP5/8 specifically localizes to PS-rich MAM subdomains.	ORP5/8 coordinates LD biogenesis and maintenance by recruiting and interacting with seipin, a key regulator of LD formation, to establish a three-way contact point between the ER, mitochondria, and LDs.
Coiled-coil domain-containing protein 127 (CCDC127)	CCDC127 localizes to mitochondria, positively regulates LDs, and is involved in the formation of MERCs.	CCDC127 is a mitochondrial protein that positively regulates LDs and promotes mito-ER contacts by regulating the protein level of VAPA. In addition, disruption of CCDC127 leads to reduced LDs and impairment in adipocyte differentiation.
Vacuolar protein sorting-associated protein 13A (VPS13A)	VPS13A is a peripheral membrane protein, associated with mitochondria, ER and LDs. VPS13A is localized at sites where the ER and mitochondria are in close contact, as well as at LDs.	VPS13A interacts with the ER residing protein VAPA via its FFAT domain. Interaction with mitochondria is mediated via its C-terminal domain. In VPS13A-depleted cells, ER-mito contact sites are decreased, mitochondria are fragmented and mitophagy is decreased. VPS13A also localizes to LDs and affects LD motility and number.
Calsyntenin-3β (CLSTN3β)	CLSTN3β is localized at ER-LD contact sites and can regulate LD-mito interactions in the liver.	CLSTN3β is an ER-LD contact site protein that was previously thought to be expressed only in adipose tissue. Recent studies have revealed its key role in hepatic metabolism. *Clstn3b* expression is induced in the liver by the nuclear receptor PPARα in settings of high lipid utilization, including fasting and ketogenic diet feeding. Hepatocyte-specific loss of CLSTN3β in mice impairs ketogenesis independent of changes in PPARα activation. Conversely, hepatic overexpression of CLSTN3β promotes ketogenesis in mice. Mechanistically, CLSTN3β affects LD-mito crosstalk, as evidenced by changes in FA oxidation, lipid-dependent mitochondrial respiration, and the mitochondrial integrated stress response. However, conclusive evidence indicating a direct interplay between CLSTN3β and specific proteins on LDs or mitochondria is lacking.

## Dysregulation of Mito-ER-LD Interactions in T2DM

mitochondria, ER, and LDs form a finely tuned network that regulates cellular metabolic homeostasis through dynamic physical contacts and functional coupling. In T2DM, this interaction network undergoes characteristic dysregulation in adipose tissue, the liver, skeletal muscle, and pancreatic β cells, collectively driving the onset and progression of lipid metabolic reprogramming and insulin resistance (Fig. 3).

**Fig. 3:**
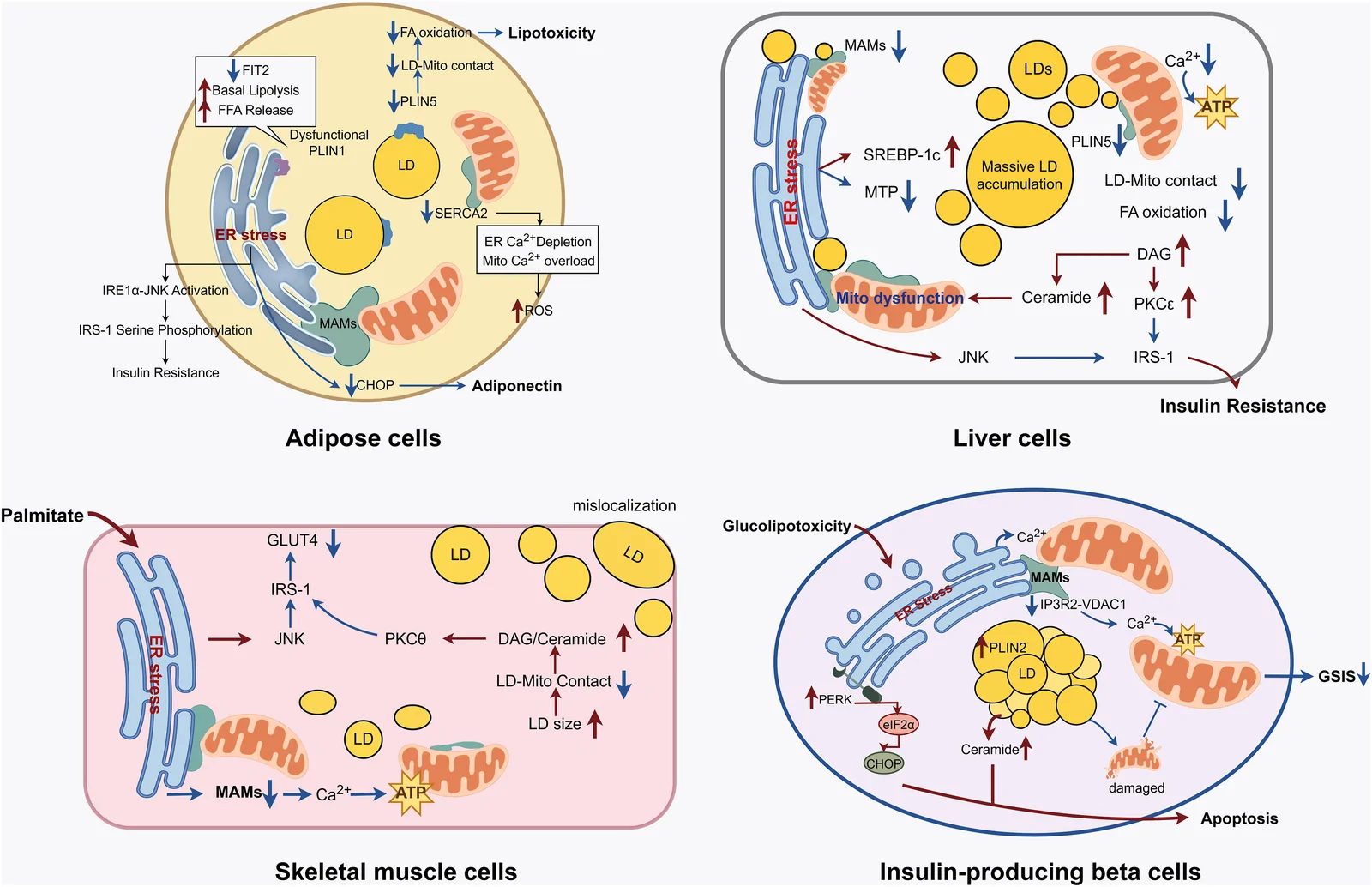
Mechanisms of organelle interactions in T2DM in various tissue cells. In adipocytes, enhanced basal lipolysis and free-fatty-acid release, defective perilipin 1, reduced lipid droplet-mitochondria contacts, and mitochondria-associated endoplasmic reticulum membrane dysfunction promote oxidative stress, impaired adiponectin secretion, and insulin resistance. In hepatocytes, endoplasmic reticulum stress, excessive lipogenesis, lipid droplet accumulation, impaired fatty-acid oxidation, ceramide and diacylglycerol accumulation, and disrupted insulin signaling reinforce mitochondrial dysfunction and hepatic insulin resistance. In skeletal muscle cells, palmitate-induced endoplasmic reticulum stress, reduced mitochondria-associated endoplasmic reticulum membranes and calcium transfer, enlarged lipid droplets, and diminished lipid droplet-mitochondria contacts impair glucose transporter type 4 trafficking and insulin action. In insulin-producing beta cells, glucolipotoxicity disrupts mitochondria-associated endoplasmic reticulum membranes and calcium signaling, promotes endoplasmic reticulum stress and lipotoxicity, and culminates in impaired glucose-stimulated insulin secretion and apoptosis. Abbreviations: ATP, adenosine triphosphate; Ca^2+^, calcium ion; CHOP, CCAAT/enhancer-binding protein homologous protein; DAG, diacylglycerol; eIF2alpha, eukaryotic initiation factor 2 alpha; ER, endoplasmic reticulum; FA, fatty acid; FFA, free fatty acid; FIT2, fat storage-inducing transmembrane protein 2; GLUT4, glucose transporter type 4; GSIS, glucose-stimulated insulin secretion; IP3R2, inositol 1,4,5-trisphosphate receptor type 2; IRE1alpha, inositol-requiring enzyme 1 alpha; IRS-1, insulin receptor substrate 1; JNK, c-Jun N-terminal kinase; LD, lipid droplet; MAM, mitochondria-associated endoplasmic reticulum membrane; Mito, mitochondria; MTP, microsomal triglyceride transfer protein; PERK, protein kinase R-like endoplasmic reticulum kinase; PKCepsilon, protein kinase C epsilon; PLIN1/2/5, perilipins 1, 2, and 5; ROS, reactive oxygen species; SERCA2, sarco/endoplasmic reticulum calcium ATPase 2; SREBP-1c, sterol regulatory element-binding protein 1c; VDAC1, voltage-dependent anion channel 1.

### Adipose tissue

White adipose tissue is not only an energy storage depot but also an active endocrine organ. Under conditions of obesity and T2DM, the mito-ER-LD interaction network in adipocytes undergoes profound remodeling and becomes an initiator of systemic insulin resistance.

#### ER-LD crosstalk and adipocyte dysfunction

Under conditions of nutrient excess, adipocytes synthesize large amounts of TAG in the ER through enzymes such as DGAT2 and store it in the form of LDs. This process depends on sustained involvement of the ER, which supplies lipid synthesis enzymes and LD-coating proteins, including PLIN1. PLIN1 is the principal LD-coating protein in adipocytes and, under basal conditions, protects LDs from hydrolysis by lipases such as ATGL (188). Once adipocyte hypertrophy surpasses the physiological capacity for expansion, the protein-folding load within the ER rises sharply and induces ER stress. ER stress activates the IRE1α-c-Jun N-terminal kinase (JNK) pathway, leading to serine phosphorylation of insulin receptor substrate 1 (IRS-1) and thereby inducing insulin resistance in adipocytes (189, 190). More importantly, mutations in the PLIN1 gene cause familial partial lipodystrophy, enhance basal lipolysis, and promote the release of large amounts of FFAs into the circulation, resulting in severe insulin resistance and fatty liver disease. In addition, the expression of FIT2 is reduced in the adipose tissue of patients with T2DM, and its downregulation impairs the TAG storage capacity of LDs while inducing ER stress and insulin resistance (191).

#### LD-mito crosstalk and energy metabolic coordination

In brown adipose tissue, cytoplasmic mitochondria exhibit higher FA oxidation capacity, whereas peridroplet mitochondria preferentially oxidize pyruvate, thereby supporting LD expansion by shifting the balance from lipolysis toward TG synthesis. Recent evidence identifies mitochondrial Ca^2+^ handling as a regulatory switch between these metabolic states. A surge in mitochondrial matrix Ca^2+^ induces detachment of PDMs from LDs, clearing the lipid droplet surface for lipolytic activity. Conversely, extrusion of Ca^2+^ by NCLX promotes attachment of mitochondria to LD. Consistent with this mechanism, PDE2A inhibition activates NCLX and increases peridroplet mitochondria abundance in the brown adipose tissue of obese mice (192).

In healthy adipocytes, LDs and mitochondria establish physical contacts through proteins such as PLIN5, thereby coordinating the storage and oxidation of FAs. Under conditions of increased energy demand, PLIN5 recruits ATGL to the LD surface, promoting local lipolysis and directly supplying the released FAs to adjacent mitochondria for oxidation (193, 194). However, in adipose tissue from T2DM, this efficient LD-mito cooperation is disrupted. Abnormal expansion of LDs leads to alterations in their surface protein composition, a reduced proportion of PLIN5 coverage, and decreased LD-mito contacts. As a result, FA oxidation is impaired, lipotoxic intermediates accumulate, and local insulin resistance is further aggravated.

#### MAMs and regulation of adipokine secretion

MAMs, which serve as a physical interface between mitochondria and the ER, regulate Ca^2+^ homeostasis and energy metabolism in adipocytes. In adipocytes from patients with T2DM, reduced expression of SERCA2, the ER Ca^2+^ pump, causes ER Ca^2+^ depletion and mitochondrial Ca^2+^ overload, thereby triggering mitochondrial ROS generation and ER stress and ultimately impairing adipokine secretion (195). Adiponectin synthesis and secretion are highly dependent on ER function. ER stress not only induces adiponectin protein degradation through the activation of autophagy but also downregulates adiponectin gene expression through transcriptional repressors such as CCAAT/enhancer-binding protein homologous protein (CHOP), thereby reducing the secretion of biologically active high-molecular-weight adiponectin (196, 197). In contrast, adipocyte-specific overexpression of spliced X-box binding protein 1 can promote adiponectin multimerization by upregulating ER chaperone proteins, thereby increasing high-molecular-weight (HMW) adiponectin secretion and improving systemic insulin sensitivity (198).

#### Dysregulation of mito-ER-LD interactions

In T2DM and obesity, adipocyte hypertrophy triggers ER stress, leading to activation of JNK and inactivation of IRS-1, ultimately resulting in insulin resistance. insulin resistance leads to dysfunction of PLIN1, enhanced basal lipolysis, and the release of large amounts of FFAs into circulation. These FFAs further aggravate ER stress and inflammation, including M1 macrophage infiltration, through receptors such as toll-like receptor 4, thereby forming a positive feedback loop (199). Meanwhile, ER stress induces autophagic degradation of adiponectin and suppresses its expression through CHOP, resulting in reduced circulating levels of HMW adiponectin. Downregulation of LD proteins such as FIT2 impairs the storage capacity of LDs and further aggravates lipotoxicity. Abnormal function of MAMs, such as downregulation of SERCA2, disrupts Ca^2+^ homeostasis and affects mitochondrial metabolism and adipokine secretion. Ultimately, adipose tissue shifts from a protective energy-storage organ to a source of pro-inflammatory and pro-lipotoxic signaling, promoting ectopic lipid accumulation in the liver and skeletal muscle.

### Liver

The liver is a central organ for systemic lipid metabolism and gluconeogenesis, and the coordination of inter-organelle interactions is severely disrupted in T2DM and non-alcoholic fatty liver disease. The mito-ER-LD interactions determine whether lipids are stored in LDs or oxidized by mitochondria.

#### ER-LD axis

In the liver of T2DM, nutrient excess, including a high-fat and high-fructose diet, leads to intracellular lipid overload in hepatocytes and triggers ER stress. The three major UPR pathways, including the PERK/eukaryotic initiation factor 2 alpha (eIF2α)/CHOP pathway, the IRE1α/X-box binding protein 1 (XBP-1)/JNK pathway, and ATF6, are all activated. JNK activated by IRE1α phosphorylates IRS-1 at Ser307, thereby inhibiting insulin signaling and inducing hepatic insulin resistance. Meanwhile, ER stress promotes hepatic de novo lipogenesis by activating sterol regulatory element-binding protein 1c (SREBP-1c) and upregulating the expression of lipogenic genes such as fatty acid synthase. Importantly, ER stress suppresses microsomal triglyceride transfer protein (MTP) activity, disrupts the assembly and secretion of VLDL, and consequently drives extensive TAG accumulation within hepatocytes as LDs, resulting in hepatic steatosis. This imbalance, characterized by excessive lipid synthesis and defective lipid export, establishes a vicious cycle that drives abnormal LD expansion (200, 201).

#### LD-mito axis

Evidence from metabolic liver disease further shows that the peridroplet mitochondria-cytoplasmic mitochondria balance is actively remodeled. For example, peridroplet mitochondria in liver varies with the severity of metabolic dysfunction associated steatotic liver disease (MASLD) (202). In db/db mice with T2DM-associated MASLD, insulin resistance increased both the number and size of lipid droplets in the liver by enhancing the accumulation of FFAs, which is accompanied by an increase in contacts with mitochondria. Both the morphology and relative contribution of cytoplasmic mitochondria- and peridroplet mitochondria-centered LD contacts were altered: in cytoplasmic mitochondria, enhanced FA oxidation resulted in elongated mitochondria that surrounded single small LDs and were responsible for LD consumption, while in peridroplet mitochondria, increased substrates for lipid synthesis promoted LD expansion with the assistance of the ER (203). The functional divergence between cytoplasmic mitochondria and peridroplet mitochondria also changes during progression from steatosis to metabolic dysfunction-associated steatohepatitis (MASH). Peridroplet mitochondria showed higher pyruvate oxidation and OXPHOS, while cytoplasmic mitochondria favored FA oxidation with MASH progression. TEM revealed larger and elongated mitochondria during healthy and early steatosis, which appeared small and fragmented during MASH progression. These changes coincided with higher MFN2 protein levels in hepatic peridroplet mitochondria and higher DRP1 protein levels in hepatic cytoplasmic mitochondria (204).

PLIN5 is an important regulator of this spatial and metabolic partitioning. Its phosphorylation controls mito-LD coupling and hepatic lipid flux, and dysregulation of this pathway promotes steatosis. Nutrient excess in T2DM may therefore disrupt the adaptive balance between lipid storage and oxidation. Excessive or persistent peridroplet mitochondria-associated lipid storage, insufficient cytoplasmic mitochondria-mediated oxidation, or defective transfer between these states can increase hepatic TAG while allowing DAG and ceramide to accumulate. These intermediates activate PKC epsilon and impair IRS-1 signaling (205, 206).

#### MAMs

MAMs play a double-edged sword role in hepatic metabolism. Under physiological conditions, MAMs facilitate the efficient transfer of ER-released Ca^2+^ into mitochondria, thereby activating key enzymes of the TCA cycle and enhancing mitochondrial metabolism and ATP production (207). However, in T2DM, signaling mediated by MAMs may become dysregulated. For instance, accumulation of specific ceramides, including ceramide (C24:0), may induce mitochondrial Ca^2+^ overload and dysfunction and further exacerbate ER stress. Evidence further indicates that ER stress increases the expression of SKIP, a phosphatidylinositol-3,4,5-trisphosphate phosphatase, thereby inhibiting insulin signaling (208). Moreover, restoring the expression of heat shock protein 72 or Nogo-B receptor may ameliorate insulin resistance and hepatic steatosis by alleviating ER stress and JNK activity (209, 210).

#### Dysregulation of mito-ER-LD interactions

In the liver of T2DM, nutrient excess induces ER stress, activates JNK, and inactivates IRS-1, thereby leading to insulin resistance. ER stress simultaneously upregulates SREBP-1c, enhances lipid synthesis, and reduces VLDL secretion by suppressing MTP, thereby causing abnormal LD accumulation. Accumulated LDs show reduced PLIN5 on their surface, disrupted LD-mito contacts, decreased FA oxidation, and accumulation of DAG and ceramide, which further activates PKCε and aggravates serine phosphorylation of IRS-1. Meanwhile, dysfunction of MAMs disrupts Ca^2+^ signaling and reduces mitochondrial ATP generation, further aggravating metabolic disturbances. Ultimately, a vicious cycle is formed in which ER stress, LD accumulation, mitochondrial dysfunction, and insulin resistance mutually reinforce one another.

### Skeletal muscle

Skeletal muscle is the principal site of insulin-stimulated glucose uptake and FA oxidation, and coordination among these three organelles is essential for maintaining metabolic flexibility. Dysregulation of this interaction network is one of the most important factors driving systemic insulin resistance.

#### LD-ER interactions

Increased circulating saturated FAs, including palmitic acid, can trigger ER stress after uptake by muscle cells (211, 212). Activation of the PERK/eIF2α and IRE1α/XBP-1 pathways upregulates pro-apoptotic factors such as CHOP and activates JNK through the IRE1α-tumor necrosis factor receptor-associated factor 2 complex (213). Activation of JNK induces phosphorylation of IRS-1 at Ser307, suppresses insulin-stimulated phosphoinositide 3-kinase (PI3K)/protein kinase B (AKT) signaling, and reduces translocation of GLUT4 to the plasma membrane, thereby decreasing glucose uptake (214). Hyperglycemia and advanced glycation end products can also activate the PERK/forkhead box O1 (FOXO1) pathway through ROS-mediated ER stress, thereby leading to muscle atrophy (215). Exercise training, caloric restriction, or specific nutrients and pharmacological agents, such as resveratrol and melatonin, can improve insulin sensitivity by activating AMPK and suppressing ER stress (216, 217).

#### LD-mito interactions

Endurance athletes exhibit high LD content in myocytes together with high insulin sensitivity, whereas patients with T2DM also display abundant intramyocellular LDs but develop insulin resistance, and the key determinants are LD morphology, subcellular distribution, and the efficiency of LD-mito coordination (218, 219). Recent human studies further show that endurance athletes have more extensive LD-mito contacts, particularly within the intermyofibrillar compartment and mitochondria-rich fibres (220). In healthy muscles, LDs are small and numerous and are predominantly located in the intermyofibrillar region, where they are tightly coupled to mitochondria through PLIN5 (221, 222). This spatial proximity facilitates the efficient transfer of FAs stored in LDs to adjacent mitochondria for β-oxidation. PLIN5 can bind both LDs and mitochondria and recruit ATGL, thereby promoting local lipolysis and channeling FAs directly to mitochondria for oxidation. In the skeletal muscle of patients with T2DM, LDs are enlarged and are predominantly located in the subsarcolemmal region, distant from mitochondria (218). Although total PLIN5 expression may not be decreased, fewer LDs are covered by PLIN5, whereas PLIN2 may be more abundant on the LD surface (223, 224). Increased abundance of PLIN2 is associated with decreased recruitment of ATGL to LDs, leading to impaired lipolysis. Owing to reduced LD-mito contacts and dysregulated lipolysis, the capacity for FA oxidation declines, leading to accumulation of DAG and ceramide in the cytoplasm (206, 225). DAG can activate protein kinase C theta (PKCθ), thereby directly inhibiting the function of IRS-1.

#### Interactions at MAMs

In animal models of obesity and T2DM, as well as in the skeletal muscle of patients, the integrity of MAMs is impaired, representing an early event that precedes mitochondrial dysfunction and overt insulin resistance (226). Palmitic acid treatment can directly reduce the number of MAMs in human myotubes. More importantly, artificial enhancement of MAMs through genetic manipulation can protect myocytes from palmitic acid-induced insulin resistance, whereas disruption of MAMs impairs insulin signaling. This indicates that the integrity of MAMs is essential for maintaining insulin sensitivity in skeletal muscle.

#### Dysregulation of mito-ER-LD interactions

In skeletal muscle from T2DM, palmitic acid triggers ER stress and activates JNK, leading to serine phosphorylation of IRS-1. At the same time, LD-mito cooperation fails, as LDs enlarge and become distant from mitochondria, PLIN5 coverage decreases, LD-mito contacts are disrupted, and the capacity for FA oxidation declines. Unoxidized FAs are converted into DAG and ceramide, accumulate in the cytoplasm, and activate PKCθ, thereby further inhibiting IRS-1. Disruption of MAMs integrity leads to reduced mitochondrial Ca^2+^ uptake and insufficient ATP production, thereby aggravating insulin resistance and muscle dysfunction. Exercise can reverse this imbalance by activating AMPK, promoting mitochondrial biogenesis, enhancing LD-mito contacts, and improving the function of MAMs.

### Pancreas

Pancreatic β cells are the only cells that produce insulin, and their highly developed ER system renders them particularly sensitive to ER stress.

#### LD-ER interactions

Under physiological conditions, LDs in β cells can serve as a temporary “buffer pool” for FAs, storing excess FFAs in the form of neutral lipids and preventing their entry into harmful metabolic pathways. In T2DM, persistent exposure to a high-lipid environment causes the influx of FFAs to exceed the storage and turnover capacity of LDs, resulting in abnormal LD enlargement and accumulation. Expression of PLIN2, a LD-coating protein, is markedly upregulated in β cells from patients with T2DM, and this is associated with intracellular lipid accumulation and impaired autophagic function in β cells (227). Once the protective storage capacity of LDs becomes saturated, FFAs are diverted into non-oxidative pathways, generating lipotoxic mediators such as ceramides, which directly impair mitochondrial function, induce ER stress, and activate apoptotic pathways (228).

#### MAMs

Glucose-stimulated insulin secretion (GSIS) depends on Ca^2+^ signal amplification mediated by MAMs. Glucose metabolism elevates the ATP/ADP ratio, resulting in closure of ATP-sensitive potassium (K-ATP) channels, plasma membrane depolarization, opening of voltage-gated Ca^2+^ channels, and Ca^2+^ influx. The influx of Ca^2+^ induces Ca^2+^ release from the ER via IP3Rs, a process known as Ca^2+^-induced Ca^2+^ release (229). The released Ca^2+^ efficiently enters adjacent mitochondria through MAMs, mediated by the IP3R-GRP75-VDAC1 complex, rapidly activates key dehydrogenases in the TCA cycle, markedly increases ATP production, and provides sufficient energy for the exocytosis of insulin granules (230, 231). In β cells from patients with T2DM, the structural integrity of MAMs is impaired, and the IP3R2-VDAC1 complex is reduced, leading to decreased efficiency of ER-mito Ca^2+^ transfer, weakened amplification of GSIS signaling, and ultimately insufficient insulin secretion (232).

#### ER stress and the UPR

β cells must rapidly synthesize, fold, and secrete large amounts of insulin in response to fluctuations in blood glucose, placing ER function under highly dynamic and stressful conditions. Under conditions of insulin resistance, β cells must compensatorily secrete more insulin, resulting in excessive ER protein-folding burden and triggering ER stress. Moderate UPR activation aims to restore ER homeostasis by upregulating ER chaperone proteins, promoting ER-associated degradation, and transiently reducing protein synthesis. However, when ER stress is prolonged or overwhelming, the UPR switches from adaptive signaling to the initiation of apoptosis. The PERK-eIF2α-ATF4-CHOP axis is a key mediator of β-cell apoptosis, as CHOP increases the expression of pro-apoptotic proteins, including p53 upregulated modulator of apoptosis, while suppressing anti-apoptotic proteins such as B-cell lymphoma 2, thereby ultimately triggering the caspase cascade (233, 234, 235). Multiple factors can induce ER stress in β cells, including lipotoxicity mediated by saturated FAs, glucotoxicity, inflammatory cytokines such as interleukin-1 beta (IL-1β), and genetic mutations, including mutations in the insulin gene, Wolfram syndrome 1 gene, and PERK (236, 237, 238, 239, 240).

#### Imbalance of mito-ER-LD interactions

In healthy β cells, ATP generated by glucose metabolism closes K-ATP channels, thereby triggering Ca^2+^ influx and ER Ca^2+^ release, after which Ca^2+^ enters mitochondria through MAMs to amplify ATP signaling and drive insulin secretion. LDs serve as temporary reservoirs for FFAs, thereby protecting cells from lipotoxicity. In T2DM, genetic or environmental factors, such as lipotoxicity, induce ER stress and activate the CHOP-mediated pro-apoptotic pathway. Meanwhile, loss of integrity of MAMs abolishes effective amplification of GSIS signaling. When the protective capacity of LDs becomes saturated, excess FFAs are diverted to generate lipotoxic mediators such as ceramides, which activate JNK and further impair insulin signaling and mitochondrial function. Defective autophagy results in the accumulation of damaged mitochondria and misfolded proteins, which further exacerbates ER stress and oxidative injury. Ultimately, β cells progress from functional compensation to dysfunction and apoptosis, leading to absolute insulin secretory insufficiency and driving T2DM from the stage of insulin resistance into the decompensated stage.

## Effects of clinical drugs and lifestyle interventions on Mito-ER-LD interactions

With the deepening understanding of the pathogenesis of T2DM, the mito-ER-LD interaction network has emerged as an important target for therapeutic intervention. Various glucose-lowering drugs and lifestyle interventions can improve insulin resistance, protect β-cell function, and alleviate lipotoxicity and systemic metabolic disorders by regulating the functions of these three organelles and their interactions (Fig. 4).

**Fig. 4:**
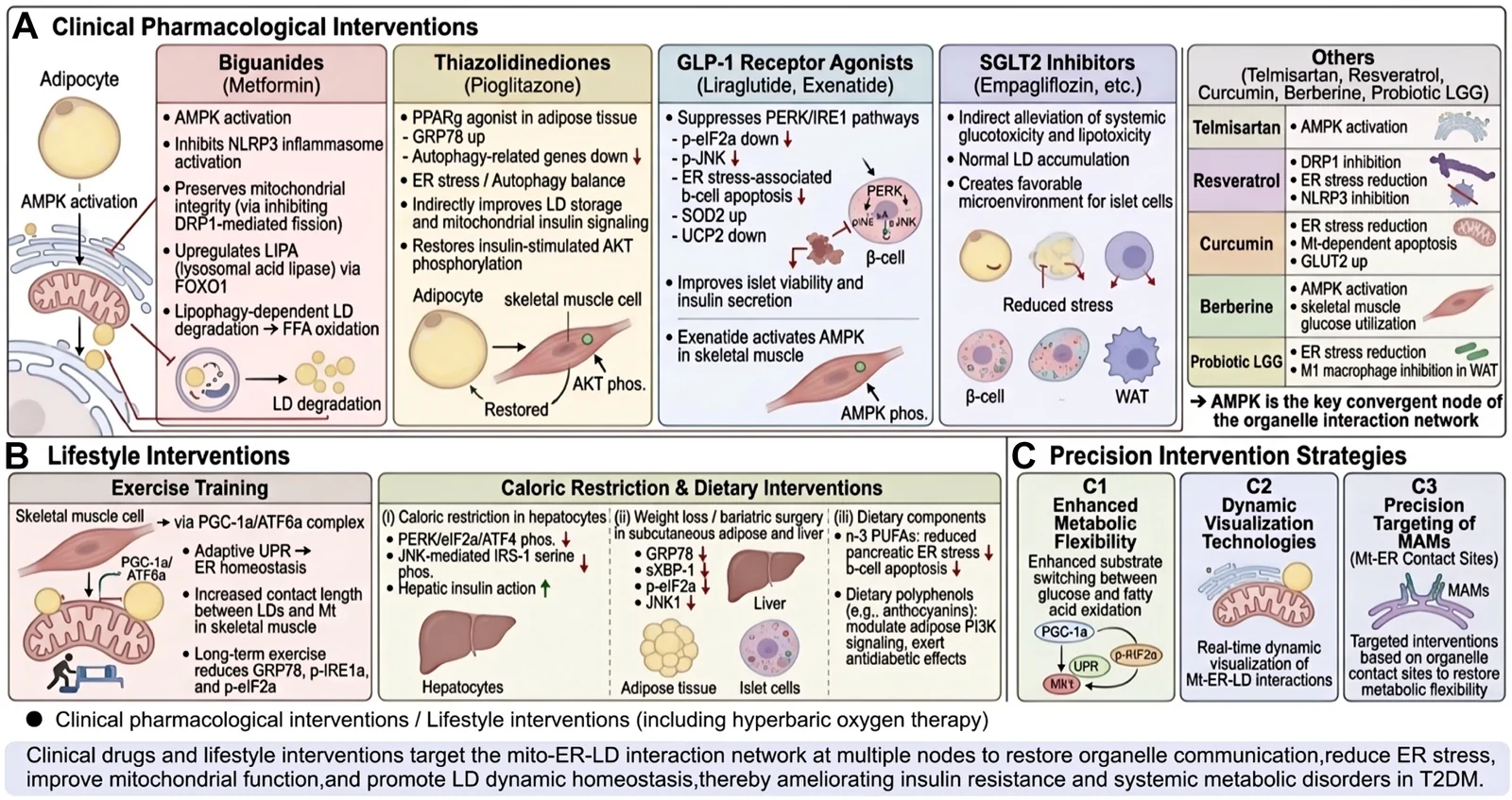
Pharmacological, lifestyle, and precision interventions targeting mitochondria-endoplasmic reticulum-lipid droplet interactions in type 2 diabetes mellitus. A, metformin, pioglitazone, glucagon-like peptide-1 receptor agonists, sodium-glucose cotransporter 2 inhibitors, and other agents act at complementary nodes to improve mitochondrial integrity, lipid droplet turnover, endoplasmic reticulum homeostasis, insulin signaling, and beta-cell survival; AMP-activated protein kinase is highlighted as a convergent regulatory node. B, exercise, caloric restriction, weight loss or bariatric surgery, and dietary components remodel organelle contacts, reduce endoplasmic reticulum stress, and improve metabolic flexibility. C, proposed precision strategies include enhancing substrate switching, dynamically visualizing organelle contacts, and directly targeting mitochondria-associated endoplasmic reticulum membranes. Abbreviations: AKT, protein kinase B; AMPK, AMP-activated protein kinase; ATF4/6alpha, activating transcription factors 4 and 6 alpha; DRP1, dynamin-related protein 1; eIF2alpha, eukaryotic initiation factor 2 alpha; ER, endoplasmic reticulum; FFA, free fatty acid; FOXO1, forkhead box O1; GLP-1, glucagon-like peptide-1; GLUT2, glucose transporter type 2; GRP78, glucose-regulated protein 78; IRE1, inositol-requiring enzyme 1; IRS-1, insulin receptor substrate 1; JNK, c-Jun N-terminal kinase; LD, lipid droplet; LGG, Lactobacillus rhamnosus GG; LIPA, lysosomal acid lipase; MAM, mitochondria-associated endoplasmic reticulum membrane; Mt, mitochondria; NLRP3, NLR family pyrin domain containing 3; PGC-1alpha, peroxisome proliferator-activated receptor gamma coactivator 1 alpha; PI3K, phosphoinositide 3-kinase; PPARgamma, peroxisome proliferator-activated receptor gamma; PUFA, polyunsaturated fatty acid; SGLT2, sodium-glucose cotransporter 2; SOD2, superoxide dismutase 2; T2DM, type 2 diabetes mellitus; UCP2, uncoupling protein 2; UPR, unfolded protein response; WAT, white adipose tissue; XBP-1, X-box binding protein 1.

### Effects of clinical pharmacological interventions

#### Biguanides

Metformin activates the AMPK signaling pathway, suppresses high glucose-induced DRP1-mediated mitochondrial fission, preserves mitochondrial integrity, and reduces phosphorylation of ER stress markers, including IRE1α and eIF2α, thereby inhibiting NLR family pyrin domain containing 3 (NLRP3) inflammasome activation and adipose tissue inflammation (241). In adipocytes, metformin induces the expression of lysosomal acid lipase through upregulation of FOXO1, thereby facilitating lipophagy-dependent LD degradation and directing FFAs toward mitochondrial oxidation to preserve energy homeostasis (242). Metformin combined with phytochemicals, such as gallic acid, can synergistically reduce markers of inflammation, oxidative stress, and ER stress in diabetic rats and improve glucose metabolism (243). These results indicate that metformin enhances insulin sensitivity by coordinating mitochondrial dynamics, ER stress responses, and LD turnover.

#### Thiazolidinediones

Pioglitazone, a peroxisome proliferator-activated receptor gamma agonist, markedly alleviates pancreatic ER stress in db/db mice and reduces apoptosis of islet cells (244). In T2DM rats, pioglitazone elevates GRP78 expression in perivascular adipose tissue while downregulating autophagy-related genes, indicating that it regulates the balance between ER stress and autophagy (245). In addition, pioglitazone can reverse ER stress-induced upregulation of tribbles pseudokinase 3 and restore insulin-stimulated phosphorylation of AKT (246). Pioglitazone primarily acts on adipose tissue to coordinate the balance between ER stress and autophagy, thereby indirectly improving LD storage function and mitochondrial insulin signaling responsiveness.

#### Glucagon-like peptide-1 (GLP-1) receptor agonists

Liraglutide markedly decreases pancreatic p-eIF2α and p-JNK levels in Akita mice, suppresses the PERK and IRE1 pathways, and mitigates ER stress-associated β-cell apoptosis (247). Exenatide pretreatment reduces pancreatic IL-1β expression in brain-dead donor rats, increases superoxide dismutase 2, inhibits uncoupling protein 2, lowers transcription of CHOP and GRP78, and enhances islet viability and insulin secretion (248). In obese Zucker rats, exenatide activates AMPK signaling in skeletal muscle and improves insulin resistance (249). GLP-1 receptor agonists preserve insulin secretion by finely modulating ER-mito functional coupling and maintaining LD dynamic homeostasis in β cells.

#### Sodium-glucose cotransporter 2 (SGLT2) inhibitors

Combined treatment with empagliflozin and gamma-aminobutyric acid can reduce FFA levels in streptozotocin (STZ)-induced diabetic mice, alleviate ER stress and inflammation in pancreatic islets, and protect β-cell (250). SGLT2 inhibitors indirectly alleviate stress signaling between the ER and mitochondria in islet cells by reducing systemic glucotoxicity and lipotoxicity, thereby creating a favorable environment for normal LD accumulation.

#### Others

Telmisartan inhibits ER stress in adipose tissue of obese mice by activating AMPK. Resveratrol can inhibit DRP1-mediated mitochondrial fission and ER stress, reduce activation of the NLRP3 inflammasome, and promote mitochondrial activity in brown adipocytes. Curcumin attenuates pancreatic ER stress and mitochondria-dependent apoptosis in STZ-induced diabetic rats, while increasing GLUT2 expression (251). Berberine promotes glucose utilization in skeletal muscle and liver by activating AMPK (252). The probiotic *Lactobacillus rhamnosus* GG can alleviate ER stress in skeletal muscle, inhibit activation of M1 macrophages in white adipose tissue, and improve glucose tolerance in db/db mice (253). The various drugs described above activate AMPK to different extents while also affecting mitochondrial fission, ER stress, and activation of the NLRP3 inflammasome, suggesting that AMPK is a key convergent node in regulating the three-organelle interaction network, and that the regulatory effects of natural products also provide a modern biological explanation for traditional Chinese medicine interventions in metabolic diseases.

### Regulatory effects of lifestyle interventions

#### Exercise training

Acute exercise markedly increases the contact length between LDs and mitochondria in skeletal muscle fibers, and this effect is positively associated with the rate of FA oxidation during exercise. Long-term exercise training can reduce GRP78 expression as well as phosphorylation of IRE1α and eIF2α in subcutaneous adipose tissue of obese individuals, thereby alleviating systemic ER stress (254). Exercise training activates the adaptive UPR in skeletal muscle through the peroxisome proliferator-activated receptor gamma coactivator 1 alpha/activating transcription factor 6 alpha complex, thereby promoting restoration of ER homeostasis (255). By increasing the physical contact length between LDs and mitochondria while simultaneously activating adaptive ER responses, exercise training remodels the three-organelle interaction network at both the levels of physical connectivity and signaling communication, thereby enhancing FA oxidation capacity and metabolic flexibility in skeletal muscle.

#### Caloric restriction and dietary interventions

Caloric restriction can significantly reduce the phosphorylation levels of ER stress markers, including PERK, eIF2α, and ATF4, in the liver and adipose tissue of ob/ob mice, decrease JNK-mediated serine phosphorylation of IRS-1, and improve hepatic insulin action (256). Weight loss after gastric bypass surgery is accompanied by significant reductions in GRP78, spliced X-box binding protein 1, p-eIF2α, and JNK1 levels in subcutaneous adipose tissue and liver, confirming that weight reduction can reverse ER stress (257). Ileal interposition surgery alleviates ER stress in the liver, adipose tissue, and pancreas by increasing circulating bile acids (258). Sleeve gastrectomy can suppress ER stress and inflammatory factors in adipose tissue while increasing expression of GLUT4 (259). n-3 polyunsaturated FAs reduce pancreatic ER stress and β-cell apoptosis in KK mice, whereas a high-saturated-fat diet disrupts muscle and liver metabolism through induction of ER stress (244, 260). Dietary polyphenols, such as anthocyanins, can exert antidiabetic effects by modulating PI3K signaling in adipose tissue (261). Caloric restriction and weight-loss surgery re-establish coordinated function among the three organelles by attenuating stress signaling from the ER to mitochondria, such as JNK-mediated suppression of IRS-1, while improving normal LD storage and distribution. Dietary components may influence the structure and function of MAMs by regulating membrane lipid composition.

#### Other interventions

Hyperbaric oxygen therapy can rapidly improve insulin sensitivity in patients with T2DM, and its mechanism is associated with reducing ER stress in skeletal muscle and increasing the mitochondrial respiratory control ratio, indicating that mild oxidative stress may enhance mitochondrial function (262). Appropriate induction of mitochondrial stress can improve mitochondrial respiratory function and concurrently reduce ER stress in skeletal muscle, and this signaling crosstalk between mitochondria and ER offers a new perspective for future interventions targeting inter-organelle interactions.

In summary, current clinical pharmacological agents and lifestyle interventions can synergistically alleviate metabolic disturbances in T2DM by targeting multiple nodes within the mito-ER-LD interaction network, thereby restoring inter-organelle communication, reducing ER stress, improving mitochondrial function, and promoting LD dynamic homeostasis. Future studies should further explore dynamic visualization technologies for three-organelle interactions and precision intervention strategies based on organelle contact sites, thereby opening new avenues for the treatment of metabolic diseases.

## Conclusions and perspectives

Current evidence indicates that mito-ER-LD interactions constitute a key regulatory hub of lipid metabolic reprogramming in T2DM. Disruption of this homeostatic balance not only promotes abnormal lipid deposition, but also further induces or exacerbates a series of pathological processes, including insulin resistance, mitochondrial dysfunction, endoplasmic reticulum stress, and pancreatic β-cell injury. In the mito-ER-LD interaction network, mitochondria represent the principal site of FA β-oxidation. The ER undertakes critical roles in lipid synthesis and calcium signaling regulation, and LDs function as dynamic organelles for neutral lipid storage and mobilization, thereby contributing substantially to the maintenance of cellular lipid homeostasis. Under physiological conditions, these three organelles achieve close structural and functional coupling through membrane contact sites: the ER participates in LD biogenesis, metabolically distinct peridroplet mitochondria and cytoplasmic mitochondria can preferentially support different aspects of lipid storage and utilization depending on tissue and nutritional state, and mito-ER crosstalk integrates Ca^2+^ signaling and maintains the dynamic balance between mitochondrial energy metabolism and ER lipid synthesis, thereby cooperatively safeguarding cellular lipid homeostasis. However, under T2DM conditions, this interaction network undergoes characteristic remodeling in adipose tissue, liver, skeletal muscle, and pancreatic β cells, thereby forming a vicious cycle of “lipid metabolic disorder-organelle dysfunction-insulin resistance” that continuously drives disease progression. Accordingly, targeting critical nodes of the interorganelle interaction network to relieve ER stress, restore mitochondrial function, and promote the dynamic homeostasis of LDs may offer new conceptual foundations and potential strategies for precision intervention and therapy in T2DM.

Although the regulatory role of mito-ER-LD interactions in lipid metabolic reprogramming in T2DM has been initially clarified, substantial limitations and challenges still persist. Most evidence for cytoplasmic mitochondria/peridroplet mitochondria specialization has been derived from adipose tissue and liver, whereas direct characterization of these mitochondrial subpopulations in human T2DM tissues remains limited. It therefore remains unclear whether T2DM alters their relative abundance, metabolic specialization, contact stability and dynamics, or the capacity to dynamically repartition lipid flux between storage and oxidation. At the methodological level, current detection approaches are unable to dynamically track the transient contacts among all three organelles simultaneously. Future efforts should focus on developing low-phototoxic, multicolor live-cell probe systems integrated with deep learning-assisted three-dimensional image analysis, thereby enabling high-throughput dynamic quantification of tripartite mito-ER-LD contacts. Moreover, most studies still remain at the level of correlative description. Tissue-specific clustered regularly interspaced short palindromic repeats-associated protein 9 (CRISPR-Cas9) should be used to generate conditional knockout or knock-in animal models, combined with time-resolved multi-omics, to directly test the causal roles of key tethering proteins, such as PLIN5, MIGA2, and Seipin, in different organs. At the level of integrating tripartite interactions, current studies have largely focused on pairwise contacts, including mito-ER, ER-LD, and LD-mito contacts, whereas structural and functional analyses of simultaneous mito-ER-LD contacts remain at an early stage. Future studies should develop probes capable of simultaneously labeling all three organelles and combine them with super-resolution three-dimensional reconstruction to reveal the dynamic evolution of three-organelle contact sites and their pathological significance in the progression of T2DM. From a clinical translational perspective, no drugs that directly target organelle contact sites have yet advanced into clinical use, and efforts should therefore be directed toward developing detection technologies based on protein and lipid signatures of MAMs derived from plasma exosomes, together with small molecules or proteolysis-targeting chimera tools for the selective modulation of specific contact sites, thereby providing target-based support for future clinical trials. Finally, at the level of systems integration, T2DM is a systemic disease, and FFAs released from adipose tissue can influence organelle interactions in the liver and muscle through the circulation, but the mechanisms of inter-organ signal transmission remain unclear. The establishment of multi-organ chip models or single-cell spatial omics maps is therefore recommended to dissect the pathways by which lipid signals are transmitted across organs through organelle contact networks, thus providing a theoretical foundation for integrated intervention strategies.

In summary, future research should pursue coordinated breakthroughs across five dimensions: methodological innovation, causal validation, tripartite integration, clinical translation, and systems medicine. With the deep integration of dynamic proximity labeling, gene editing, artificial intelligence, and spatial omics, the mito-ER-LD interaction network is expected to be transformed from a concept in basic biology into a novel target for precision diagnosis and treatment of T2DM, thereby opening the way for therapies aimed at “repairing organelle communication” in metabolic diseases.

## Data availability

All data presented are contained within the manuscript.

## Conflict of interest

The authors declare that they have no conflicts of interest with the contents of this article.
